# Revisiting maternal and child undernutrition in low-income and middle-income countries: variable progress towards an unfinished agenda

**DOI:** 10.1016/S0140-6736(21)00394-9

**Published:** 2021-03-07

**Authors:** Cesar G Victora, Parul Christian, Luis Paulo Vidaletti, Giovanna Gatica-Domínguez, Purnima Menon, Robert E Black

**Affiliations:** International Center for Equity in Health, Federal University of Pelotas, Pelotas, Brazil; Department of International Health, Bloomberg School of Public Health, Johns Hopkins University, Baltimore, MD, USA; International Center for Equity in Health, Federal University of Pelotas, Pelotas, Brazil; International Center for Equity in Health, Federal University of Pelotas, Pelotas, Brazil; Poverty, Health and Nutrition Division, International Food Policy Research Institute, New Delhi, India; Department of International Health, Bloomberg School of Public Health, Johns Hopkins University, Baltimore, MD, USA

## Abstract

13 years after the first *Lancet* Series on maternal and child undernutrition, we reviewed the progress achieved on the basis of global estimates and new analyses of 50 low-income and middle-income countries with national surveys from around 2000 and 2015. The prevalence of childhood stunting has fallen, and linear growth faltering in early life has become less pronounced over time, markedly in middle-income countries but less so in low-income countries. Stunting and wasting remain public health problems in low-income countries, where 4·7% of children are simultaneously affected by both, a condition associated with a 4·8-times increase in mortality. New evidence shows that stunting and wasting might already be present at birth, and that the incidence of both conditions peaks in the first 6 months of life. Global low birthweight prevalence declined slowly at about 1·0% a year. Knowledge has accumulated on the short-term and long-term consequences of child undernutrition and on its adverse effect on adult human capital. Existing data on vitamin A deficiency among children suggest persisting high prevalence in Africa and south Asia. Zinc deficiency affects close to half of all children in the few countries with data. New evidence on the causes of poor growth points towards subclinical inflammation and environmental enteric dysfunction. Among women of reproductive age, the prevalence of low body-mass index has been reduced by half in middle-income countries, but trends in short stature prevalence are less evident. Both conditions are associated with poor outcomes for mothers and their children, whereas data on gestational weight gain are scarce. Data on the micronutrient status of women are conspicuously scarce, which constitutes an unacceptable data gap. Prevalence of anaemia in women remains high and unabated in many countries. Social inequalities are evident for many forms of undernutrition in women and children, suggesting a key role for poverty and low education, and reinforcing the need for multisectoral actions to accelerate progress. Despite little progress in some areas, maternal and child undernutrition remains a major global health concern, particularly as improvements since 2000 might be offset by the COVID-19 pandemic.

## Introduction

Despite substantial progress in reducing global poverty and food insecurity in the past 50 years, the prevalence of maternal and child undernutrition in low-income and middle-income countries (LMICs) has remained unacceptably high.^1–3^ In the past 20 years, this prevalence has led to the implementation of interventions to reduce undernutrition, especially during the crucial first 1000 days—from conception to the second birthday—that have important consequences for survival, resistance to infection, growth, and development throughout the course of life.^4–6^ The global nutrition targets endorsed by the World Health Assembly in 2012^7^ stress the need to reduce low birthweight, childhood stunting, and wasting and anaemia in women, and to increase exclusive breastfeeding in the first 6 months of life, yet progress has been slow in most LMICs.^1–3^

The important influences of early-life undernutrition, particularly in combination with later excess weight gain, have on the development of nutrition-related diseases are well described.^2,8,9^ The increase in overweight,^2,10^ concurrent with persisting undernutrition in young children, has led to the so-called double burden of malnutrition in many countries,^9^ and research has shown that both undernutrition and overweight have similar causes.^2,10^ The UN Decade of Action on Nutrition (2016-25) and the Sustainable Development Goals call for ending malnutrition in all its forms by bringing attention to early life nutrition, diet diversity, and food systems.^11,12^

It has been 13 years since the first *Lancet* Series on maternal and child undernutrition summarised the evidence on what works to reduce multiple forms of undernutrition, the associated costs, and expected benefits.^1^ 5 years later, in the second *Lancet* Series on maternal and child nutrition^2^ published in conjunction with the Nutrition for Growth Summit in London, UK, the Series was updated with the introduction of concepts of nutrition-sensitive and nutrition-specific interventions. In the lead up to the next Nutrition for Growth Summit in 2021, we have again reviewed the evidence and lessons learned from the past decade. This Series complements other *Lancet* reports addressing nutrition and the food system (EAT Commission);^13^ the obesity, undernutrition, climate change syndemic (Obesity Commission);^14^ and the double burden of malnutrition Series.^9,10^ We focus on the burden of maternal and child undernutrition, the leading risk factor for disability-adjusted life-years lost in LMICs.^15^

For each form of undernutrition, we update the evidence on burden and consequences, and summarise new insights on determinants. We review global reports and published literature on epidemiology and determinants made available since the last Series, complemented by new analyses of data from 50 LMICs from two survey time periods. We focus on data and evidence to support the achievement of the Sustainable Development Goal target 2.2,^12^ measured by progress in reducing low birthweight, suboptimal breastfeeding, stunting and wasting in children younger than 5 years, anaemia and micronutrient deficiencies in women of reproductive age, and other measures of nutritional status.

## Fetal and child growth

### Persistent burden of fetal and child undernutrition

Childhood undernutrition includes fetal growth restriction (defined as a birthweight for gestational age and sex below the 10th centile of the InterGrowth Standards^16^), stunting, wasting, underweight (defined as Z scores of length/ height-for-age and weight-for-length/height below -2, relative to the age-specific and sex-specific WHO child growth standards) and deficiencies of vitamins and minerals are associated with elevated risks of mortality.^1,2,17–19^ From now on, we refer to length/height as height, encompassing both recumbent length for children younger than 2 years, and standing height for children aged 2-5 years. Analyses of longitudinal datasets have shown that children with concurrent wasting and stunting have the highest risk of mortality, which is the product of the independent effect of each component.^20,21^ Expanding on these findings, analyses of eight cohorts focused on measures of growth failure in the first 6 months of life.^22^ The measures most strongly associated with death by age 24 months were being severely underweight (Z scores <-3; relative risk [RR] 4·8) and having concurrent moderate wasting and moderate stunting (Z scores for both indices <-2; RR 4·8); having persistent wasting, being moderately underweight, and having severe or moderate wasting or stunting were also significantly associated with the risk of death with RRs between 2 and 3·4. In children aged 6-59 months, a mid-upper arm circumference of less than 115 mm and severe wasting (Z scores <-3) were both associated with an increased risk of mortality, and are recommended by WHO for identifying severe malnutrition.^23^ As a single screening measure, mid-upper arm circumference might be superior to severe wasting because of its simplicity and greater ability to identify children at risk of death.^24,25^ Suboptimal breastfeeding practices—defined as less than 6 months of exclusive breastfeeding, and less than 2 years of total breastfeeding—are also associated with elevated risks of death from infectious diseases as confirmed in other reviews.^26,27^

Over time, concerns about the short-term consequences of child undernutrition on morbidity and mortality have evolved to also cover lifelong effects on non-communicable diseases. The Barker hypothesis generated a huge amount of literature on how early-life undernutrition and rapid weight gains later in childhood help shape cardiovascular and metabolic health in adulthood.^8,28–30^ Associations between early nutrition and human capital—a concept that includes skills, health, knowledge, and resilience^31^—have been repeatedly supported by cohort and intervention studies. ^32,33^ Such research findings have been influential in focusing international policies on investments for pregnant women and young children.

### Stunting, wasting, and linear and ponderal growth

Global agencies estimate that stunting prevalence among children younger than 5 years declined from 32·5% in 2000 to 21·9% in 2017,^34^ yet reductions were higher in Asia (38·2% to 22·7% over the same period) and in Latin America and the Caribbean (16·7% to 9·0%) than in Africa (38·0% to 30·0%). Wasting was reduced globally from 10% in 2005^1^ to 7·3% in 2017.^34^ Levels and trends are typically described for world regions, yet there are important between-country differences in each region and within-country socioeconomic inequalities.^35^

**Figure 1:**
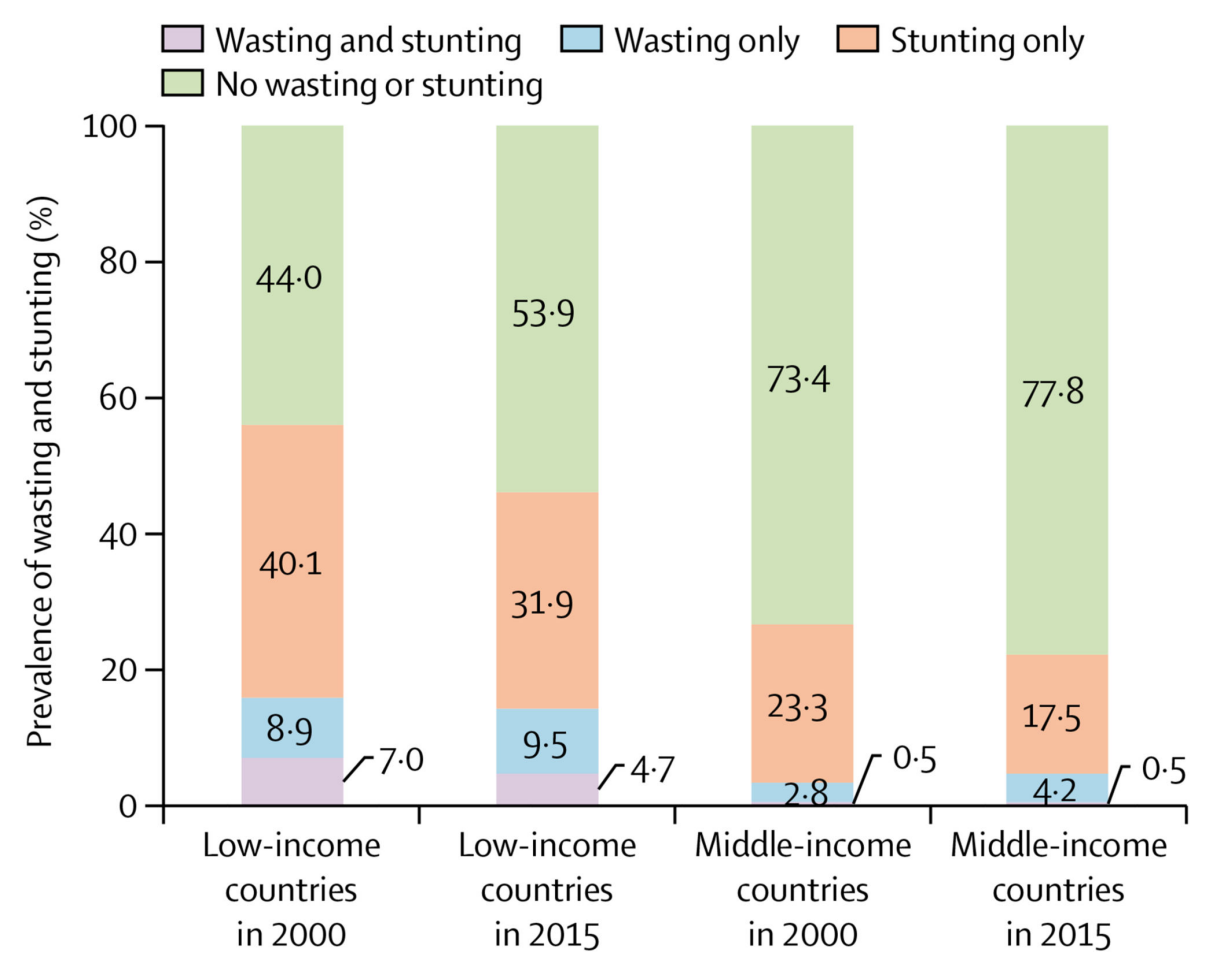
Prevalence of wasting and stunting in children younger than 5 years Data are for 31 low-income countries and 19 middle-income countries, taken from Demographic and Health Surveys and Multiple Indicator Cluster Surveys (appendix pp 1-5). We refer to data collected from 1996 to 2005 as 2000 data, and data collected from 2010 to 2018 as 2015 data.

We used national survey data from 50 LMICs from two time periods (one from 1996 to 2005, which we refer to as 2000 data, and the other from 2010 to 2018, which we refer to as 2015 data) to further explore anthropometric changes over time (for methods and data sources see appendix pp 1-5). Results are shown according to the World Bank country income groups in the year of the first survey for each country and weighted by the number of children younger than 5 years in each country. These analyses show important differences between low-income countries and middle-income countries, although there is marked variability among individual countries within each income group (appendix p 6). The moderate declines over time in stunting prevalence (figure 1) are consistent with global estimates.^34^ There was a small reduction (from 15·9% to 14·2%) in wasting prevalence in low-income countries, and a slight increase (from 3·3% to 4·7%) in middle-income countries.

**Figure 2:**
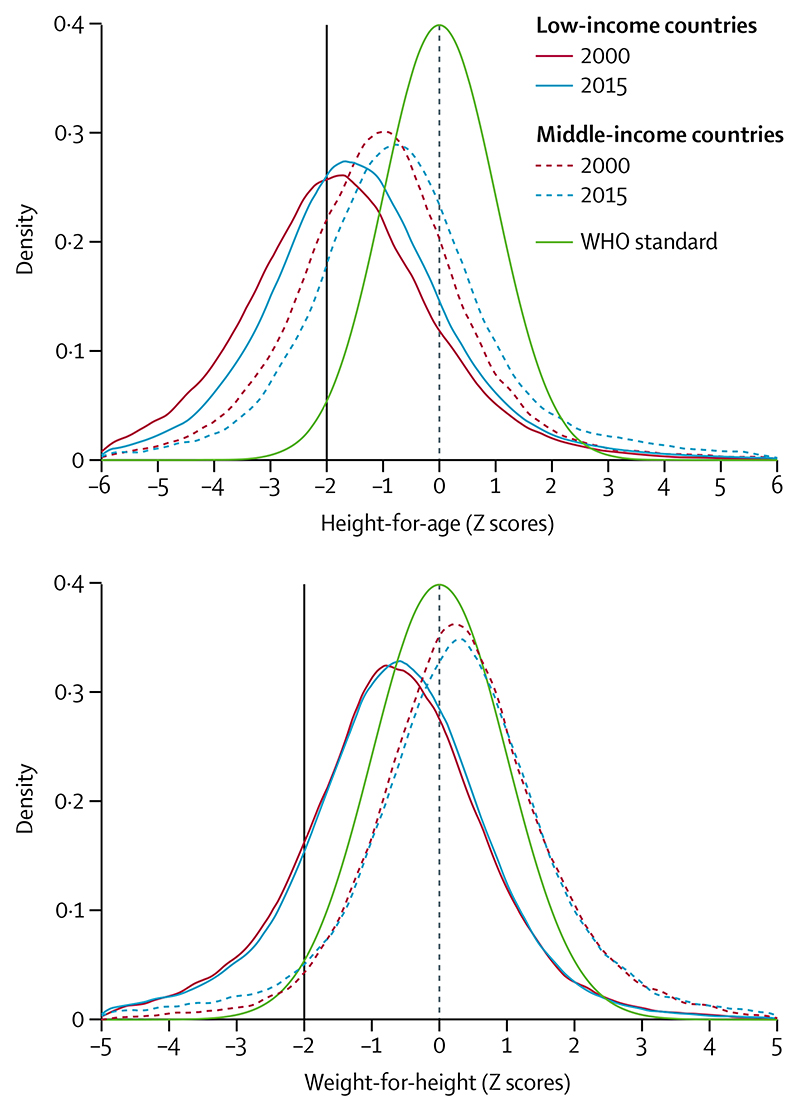
Height-for-age and weight-for-height distributions of children younger than 5 years, from 2000 and 2015 The green curves show the WHO child growth standards. The black vertical lines correspond to the traditional cutoff Z scores of −2. Data are for 31 low-income countries and 19 middle-income countries, taken from Demographic and Health Surveys and Multiple Indicator Cluster Surveys (appendix pp 1-5). We refer to data collected from 1996 to 2005 as 2000 data, and data collected from 2010 to 2018 as 2015 data.

**Figure 3:**
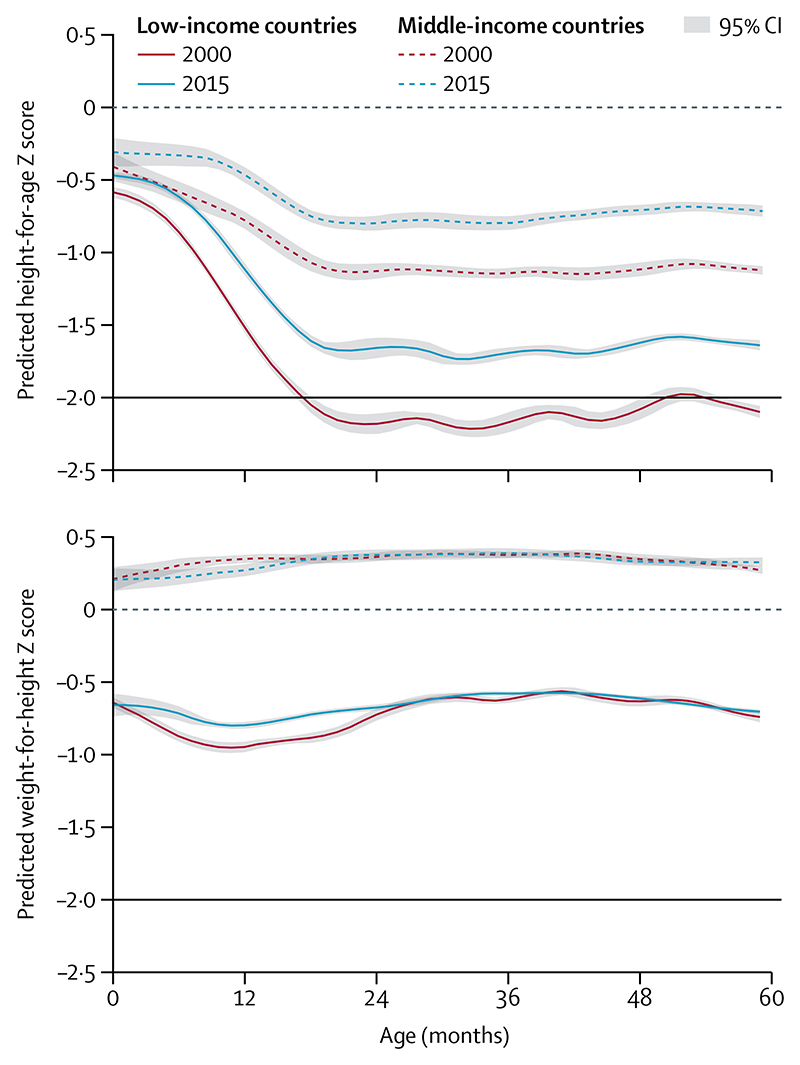
Mean height-for-age and weight-for-height Z scores by age of children younger than 5 years, from 2000 and 2015 The dashed horizontal lines at 0 represent the WHO child growth standards. The black horizontal lines correspond to the traditional cutoff Z scores of −2. Data are for 31 low-income countries and 19 middle-income countries, taken from Demographic and Health Surveys and Multiple Indicator Cluster Surveys (appendix pp 1-5). We refer to data collected from 1996 to 2005 as 2000 data, and data collected from 2010 to 2018 as 2015 data.

In low-income countries, the whole distributions of height-for-age and weight-for-height are shifted to the left of the WHO standards (figure 2), and thus undernutrition affects a large proportion of the child population, not only those below the Z score cutoff of −2.^36,37^ In middle-income countries, height-for-age curves are also shifted to the left, whereas weight-for-height curves are shifted to the right, consistent with the growing prevalence of child overweight.^9,10^

figure 3 shows mean values of height-for-age and weight-for-height Z scores from birth to age 5 years relative to WHO’s median for country income groups. The figure expresses the timing of growth faltering on the basis of Z scores, a relative measure. Absolute faltering, expressed in height (cm), continues to occur after the age of 2 years, although most of the deficit in height present at age 5 years is accrued in the first 1000 days.^38,39^ For both low-income countries and middle-income countries, there was less linear growth faltering, expressed as height-for-age, in 2015 than was observed in 2000. In low-income countries, mean birth length increased over time, and the duration of linear growth faltering was shortened. There was little change in size at birth in middle-income countries; population-level faltering started in the first month of age and continued for approximately 20 months in 2000, whereas in 2015, it started between the ages of 6 and 12 months and continued until about 18 months.

Patterns for weight-for-height faltering were vastly different from those observed for linear growth. In low-income countries, children were born thin and—for data from 2000—mean weight-for-height continued to falter relative to the standard until about age 9 months, with a slight improvement up to about age 30 months followed by a slight decline up to 60 months. The curves from 2015 are similar, except that there is little faltering from birth to age 2 years. In middle-income countries, mean Z scores are higher than WHO’s standard at all ages in both years, with slight increases from birth to about age 24 months, being stable thereafter. For both indices, children from middle-income countries were much taller and heavier at all ages than those from low-income countries. Yet, there is substantial variability among countries within each income group (appendix pp 3-6).

**Figure 4:**
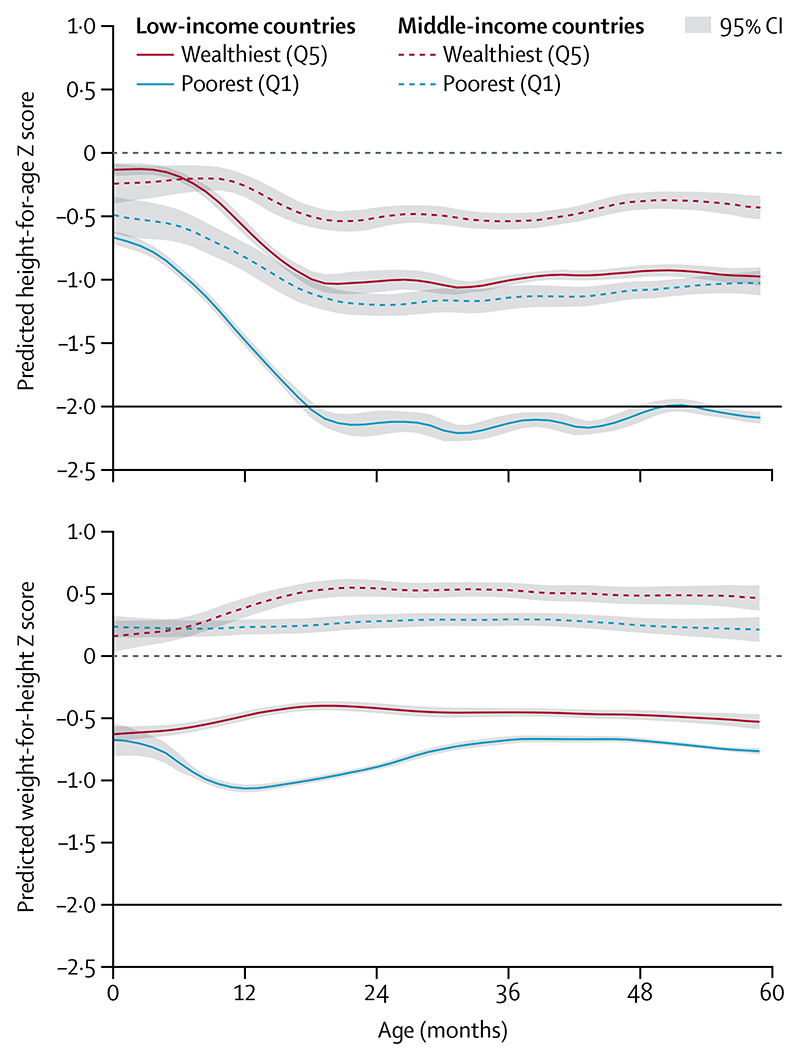
Mean height-for-age and weight-for-height Z scores by age of children younger than 5 years in the poorest and wealthiest quintiles in 2015 The dashed horizontal lines at 0 represents the WHO child growth standards. The black horizontal lines corre-spond to the traditional cutoff Z scores of −2. Data are for 31 low-income countries and 19 middle-income countries, taken from Demographic and Health Surveys and Multiple Indicator Cluster Surveys (appendix pp 1-5). We refer to data collected from 1996 to 2005 as 2000 data, and data collected from 2010 to 2018 as 2015 data. Q1=quintile 1. Q5=quintile 5.

The 2015 results are further disaggregated by household wealth (figure 4; appendix pp 7-8). In low-income countries, there are important differences in birth length between children from wealthy and poor backgrounds. Mean values of height-for-age suggest that children from poor households also present more intense and longer periods of linear growth faltering than those from wealthier backgrounds. The differences in middle-income countries follow a similar pattern to those in low-income countries, but are less marked. Weight-for-length at birth was similar for children from wealthy and poor backgrounds in low-income countries; faltering was only observed for children from the poorest quintile up to age 12 months. In middle-income countries, children from both the poorest and wealthiest quintiles were born with mean weight-for-height that was slightly higher than the WHO standard, but although mean values for children from poor house-holds remained parallel to the standard, those from the wealthiest quintile continued to increase during the first 2 years, which is consistent with growing prevalence of child overweight.

Recent studies have shown the importance of the first months of life on individual-level growth from birth to age 2 years in LMIC cohorts.^22,40^ The highest incidence of stunting was in the first 3 months of life, including 12% of babies who were already stunted at birth and 17% who had incident stunting between birth and age 3 months, accounting for 40% of those who were stunted at age 24 months. Reversal of stunting in this period was infrequent and not sustained. A similar set of cohort analyses showed that the incidence of wasting was also highest from birth to age 3 months, yet with important regional differences.^41^ In south Asia, 19% of babies had wasting at birth compared with 8% of babies in Africa and 2% in Latin America. In the first 3 months, the incidence of wasting in babies was 26% in south Asia compared with 9% in Africa and 3% in Latin America. Most children recovered from incident wasting but many had subsequent episodes resulting in incidence rates being nearly five times the highest prevalence (7%) at any age in the first 2 years of life.^41^ Wasting was also highly seasonal with the lowest weight-for-length Z scores during the rainy season, particularly in south Asia.

Because of its large population and high prevalence, children in India accounted for a large share of the global burden of wasting and stunting. Comparisons of India with other low-income countries show small differences in stunting, but markedly higher prevalence of wasting. From 1998 to 2015, height-for-age improved in India, but there was no progress in terms of weight-for-height (appendix pp 9-14).

### Concurrence of stunting and wasting

The prevalence of concurrent stunting and wasting has received increased attention over the past decade.^42^ Children are at particularly high risk of mortality, even higher than the risk in children with severe wasting (Z scores <-3).^18,21^ In an analysis of survey data from 84 countries, the overall prevalence of combined stunting and wasting was 3·0% in surveys done between 2005 and 2015 and the highest prevalence (4·4%) was in south Asia.^43^ In nine countries, concurrent wasting and stunting prevalence was higher than 5% among children aged 6-59 months. The highest prevalence of concurrent stunting and wasting was at ages 12-35 months, age groups in which stunting prevalence is typically high. Cohort analyses show that the peak prevalence of concurrent wasting and stunting at age 2 years was much higher in south Asia (8%) than in Africa (2%) or Latin America (1%).^41^ Although wasting and stunting share common determinants in utero and in infancy, there is evidence that wasting increases the risk of subsequent stunting, suggesting that the body responds to weight faltering by slowing linear growth.^44,45^

Our 50-country time trend analyses found a reduction from 7·0% (in 2000) to 4·7% (in 2015) in the prevalence of concurrent wasting and stunting in countries classified as low income in the year of the first survey, whereas in middle-income countries this prevalence remained low at 0·5% in both years (figure 1). Statistics on the concurrence of stunting and wasting were not shown in the earlier *Lancet* Series^1,2^ or in the UN joint malnutrition estimates.^34^

### Low birthweight

Low birthweight, defined as weight that is less than 2500 g in a livebirth, is associated with elevated risks of child mortality, stunting, and developmental delays, and adult-onset metabolic diseases.^33^ The two main underlying causes are being born too small (small-for-gestational age [SGA]), too soon (defined as preterm birth [gestational age <37 weeks]), or both. Annually, 23·3 million babies are estimated to be born SGA^17^ and 14·8 million are preterm.^46^ The World Health Assembly proposed a target of a 30% reduction in the number of low birthweight births between 2012 and 2025.^7^ Published estimates indicate that the global prevalence of low birthweight declined from 17·5% (22.9 million) to 14·6% (20·9 million) from 2000 to 2015, an average annual reduction rate of 1·23%, less than half of the rate needed to reach the target (2·74%).^47^ Although the rate of reduction is the fastest in south Asia, more than a quarter (26·4%) of births were low birthweight in this region in 2015. Progress in maternal nutrition and health must be accelerated to reach the global target.^47^

### Determinants of undernutrition

The previous *Lancet* Series recognised that determinants of stunting and wasting include maternal nutrition, postnatal diet, disease, and nurturing care, which are, in turn, affected by distal socioeconomic and political factors.^1,2,48^ New insights on determinants affirm this framework while shedding new light on the crucial role of maternal nutrition. Short maternal height and low body-mass index (BMI) are associated with lower height-for-age and weight-for-height at age 24 months,^22^ probably mediated by small size at birth.^1,2,48^ SGA is associated with at least a fifth of childhood stunting in LMICs and with up to a third in south Asia, and preterm birth also contributes to stunting.^49^ Although experimental evidence on the effect of breastfeeding promotion on growth is not clear cut,^27^ complementary feeding interventions have shown small but statistically significant effects.^50^

Community-based cohort studies in seven LMICs have not found effects of diarrhoea or acute lower respiratory infections on growth; however, these studies showed that a higher prevalence of enteropathogens in non-diarrhoeal stools, and inflammation, were associated with reduced linear growth by age 24 months.^51^ Current interest has expanded from the effects of specific infectious diseases to the possible role in growth faltering of environmental enteric dysfunction.^52^ This condition is characterised by increased intestinal permeability, intestinal and systemic inflammation, malabsorption, changes in growth-related hormones, and intestinal microbiota dysbiosis, and is associated with living in an environment with heavy faecal contamination.^53^ In contrast, trials of low-cost water and sanitation interventions did not show an effect on child growth.^54,55^

Studies in the past decade have supported the role of social determinants, including maternal education, paternal education, household assets, and early marriage on the prevalence of stunting, wasting, and other forms of malnutrition.^56–58^ The importance of socioeconomic drivers is depicted in figures 1-4 and appendix pp 7-8, which show the anthropometric distributions for children from poor and wealthy families in 50 LMICs. In addition to driving within-country inequalities, socio-economic factors also account for a large proportion of between-country variability in undernutrition.

New research is also beginning to highlight how so-called commercial determinants—such as the growing marketing and sales of formula milk^59^ and unhealthy industrialised foods—affect childhood undernutrition by displacing healthy foods.^3,9,60^ In LMICs, young children are increasingly exposed to unhealthy foods, including fast foods and sugar-sweetened beverages.^58^

## Micronutrient deficiencies among children

Research and programmes to tackle micronutrient deficiencies in children have largely focused on vitamin A, zinc, iron, and iodine, although other deficiencies of B vitamins might also exist. The high prevalence of micronutrient deficiencies in LMICs is due to inadequate consumption of nutritious complementary foods and excess losses due to infectious morbidity such as diarrhoea. Vitamin A deficiency, associated with blindness and increased risk of death from infectious diseases, remains a problem in LMICs,^2^ with prevalence decreasing from 39% in 1991 to 29% in 2013.^61^ Although deficiency decreased in east Asia, it remained high in south Asia at 44% and in sub-Saharan Africa at 48%. The primary intervention in LMICs has been periodic high dose vitamin A supplementation, which has been shown to reduce child mortality despite having only a short-term effect on serum retinol concentration.^2^ The high prevalence of vitamin A deficiency in LMICs is due to inadequate consumption of foods with preformed vitamin A or precursors for synthesis of vitamin A in the body.

Zinc deficiency, associated with reduced linear growth and increased infectious morbidity in childhood and increased risk of premature birth, is prevalent in LMICs.^2^ Surveys in sub-Saharan African countries have generally found that more than half of the children have serum zinc concentrations less than the thresholds that indicate deficiency, as do some countries in Asia, such as Bangladesh, Cambodia, and Vietnam.^62^

WHO estimates that the global prevalence of anaemia in children was 41.7% as of 2016.^63^ Iron deficiency is a major cause of childhood anaemia. By differing methods of analysis iron deficiency might account for as much as about 42%^64^ or as little as about 25%^65^ of all anaemia cases. In endemic areas, malaria and helminths are important determinants, as are other causes of inflammation. Studies have found that, although inflammation was associated with anaemia, concomitant iron-deficiency also existed.^66^ Data are needed for populations with high rates of anaemia to understand the role of its many possible causes.

In seven LMIC cohorts, dietary adequacy for vitamins A, D, and E, folate, calcium, iron, and zinc was generally low.^67^ Successful universal salt iodisation programmes have largely eliminated severe iodine deficiency, and concern has shifted to 25 countries with mild to moderate deficiency that might lead to neurobehavioural effects.^68^

## Breastfeeding practices

In the past 20 years, evidence on the benefits of breast-feeding has accumulated. Systematic reviews suggest that in addition to well known protection against many childhood infections,^69^ longer duration and exclusivity of breastfeeding are associated with higher intelligence, lower risk of overweight and diabetes, and, possibly, a lower risk of leukaemia and type 1 diabetes.^27^ For nursing mothers, the effects include birth spacing and reduced incidence of breast and ovarian cancers.^27^ Optimal breastfeeding practices would result in the prevention of 820 000 child deaths and 20 000 breast cancer deaths each year.^27^ New knowledge is being rapidly accrued on the role of breast milk in the infant’s microbiome^70^ and epigenome,^71^ raising the possibility that additional benefits might become evident. Global analyses show a gradual increase in the prevalence of exclusive breast-feeding, but about a half of infants younger than 6 months who should be exclusively breastfed are also fed with other foods or fluids.^27,72^ The proportion of children aged 12-15 months who are breastfed has been stable at around 70% globally,^27^ whereas just over half of all world’s children are still breastfed at age 2 years.^72^ Within LMICs, breastfeeding is one of the few healthy behaviours that are more frequent in poor households than in rich households.^27,59,72^

## Undernutrition among women

Multiple forms of undernutrition among girls and adolescents lead to poor reproductive and other health consequences for women. In addition, they exacerbate the risk of poor birth outcomes, such as fetal losses, fetal growth restriction, and preterm delivery, as well as early life undernutrition and poor cognitive development among offspring.

### Underweight and short stature in women

Underweight, defined as BMI (weight-for-height squared) less than 18·5 kg/mg^2^, is common among women of reproductive age (15-45 years) in LMICs, as a result of inadequate dietary intake, illness, or both. Another indicator of intergenerational and chronic undernutrition is short stature, defined with cutoffs ranging between 145 cm and 155 cm. The consequences of underweight and short height include reduced work productivity and increased risks of maternal morbidity and poor birth outcomes, including low birthweight.

**Figure 5:**
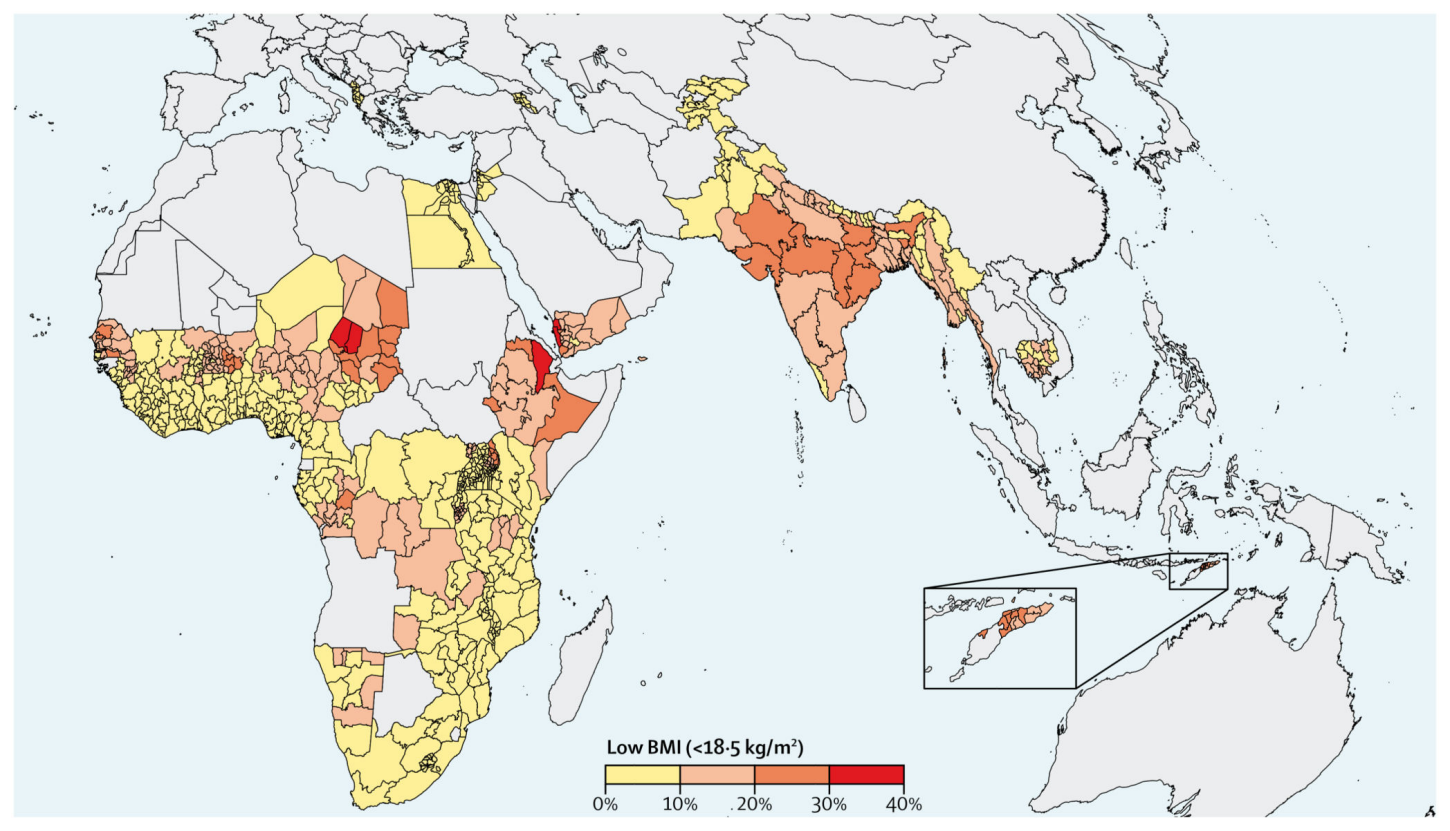
Map of low BMI (<l8.5 kg/m^2^) prevalence in women aged 15−49 years by subnational regions in African and Asian countries with available data from 2010 or later Data are taken from Demographic and Health Surveys and Multiple Indicator Cluster Surveys (appendix pp 1-5). BMI=body-mass index.

The prevalence of low BMI among women has declined globally from 14·6% in 1975 to 9·7% in 2014.^73^ Globally, age-standardised mean BMI has increased from 22·1 kg/m^2^ to 24·4 kg/m^2^. Although prevalence in 2014 was low in many parts of the world, the burden of low BMI in west, east, and central Africa, and in Asia, remains elevated, particularly in south Asia at 24%. India ranked first in 2014 in prevalence (42%), with over a 100 million women being underweight. In addition to underweight afflicting some of the poorest nations of the world, prevalence is inversely related to wealth within most LMICs.^74^ WHO recommends antenatal balanced energy and protein supplementation in settings where the prevalence of maternal low BMI (<18·5 kg/mg^2^) is 20% or more);^75^ at the national level, only India and Bangladesh would be eligible for supplementation. We undertook a subnational analysis and found numerous geographical hotspots in south Asia, and parts of sub-Saharan Africa, where the prevalence of low BMI was more than 20% (figure 5). Geographical targeting in high burden populations would result in a greater effect and probably an increased cost-effectiveness than untargeted intervention coverage,^76^ while recognising that different subgroups of women within a country might be underweight or face an increasing burden of overweight. Our subnational analysis calls for targeted interventions against the determinants of underweight to narrow the gap. Instead of BMI, maternal mid-upper arm circumference, given the ease of its use, has the potential for both screening high risk women in need for targeting and as an indicator of wasting in pregnancy.^77^

Maternal height is another strong predictor of maternal and reproductive health outcomes. In south and southeast Asia, height less than 150 cm affects as many as 40-70% of all women.^78^ Nutrition and environmental factors throughout life, especially in the first 1 000 days, but also during adolescence, influence linear growth and attained stature in adulthood.^8,79^ Over a century, there has been virtually no change in height of populations in some countries in Africa and in south and southeast Asia.^80^ A socioeconomic differential exists; however, age-standardised mean population height in low-income countries or low-wealth quintiles has remained constant, but has increased over time in upper middle-income and high-income countries or high-wealth quintiles.^79,81^ We examined prevalence trends of low BMI and low height among girls and women in 40 LMICs (table) using data from the two survey time periods referred to previously, 2010 and 2015 (appendix pp 15-17). Prevalence of low BMI has remained unchanged among girls aged 15-19 years but has halved among adult women in LMICs. Trends in the prevalence of low height-for-age (Z scores <-2) in girls and low stature (<145 cm) in women suggest some improvements, somewhat different than the published trends, although those were in the mean change over the past several decades. The higher prevalence of low height among girls (22·7%) than in adult women might be a function of the more stringent cutoff of height less than 145 cm used in adults (table). The risk of SGA and preterm births increases across height categories less than 155 cm (compared with ≥155 cm) and about 6.5 million SGA or preterm births are associated with maternal short stature annually.^78^

Suboptimal gestational weight gain, for which few data exist for LMICs, is another strong predictor of poor pregnancy outcome. Monitoring of weight gain in pregnancy or assessment of prepregnancy BMI is rarely done in LMICs. New analyses of modelled data from Demographic and Health Surveys estimate total gestational weight gain to be low in sub-Saharan Africa, north Africa and the Middle East, and south Asia, ranging between 6-7 kg,^82^ almost half the Institute of Medicine’s minimum recommendations^83^ of 11·5 kg for normal weight and 12.5 kg for women who are underweight. The availability of international fetal growth references^16^ provides the possibility of ultrasound-based assessment of fetal growth restriction, but implementation research is needed for deployment of such technologies for LMICs. Novel technologies, including handheld point-of-care medical imaging technologies, could be revolutionary.

### Anaemia in women

Anaemia during pregnancy is associated with a risk of increased morbidity and mortality,^84^ and adverse birth outcomes.^85^ The prevalence of anaemia among women of reproductive age has increased slightly from 31·6% in 2000 to 32·8% in 2016, while declining slightly among pregnant women from 41·6% to 40·1% during the same period.^86^ An analysis of repeated cross-sectional data between 2000 and 2014 found annualised absolute decreases ranging from 0.5 to a substantial 2.6 percentage points in prevalence of anaemia in women of reproductive age in 17 of 25 LMICs, although socioeconomic inequalities persisted in many settings.^87^ Data are sparse on the causes of anaemia, which varies by setting.^88^ Iron deficiency is considered a predominant nutritional cause and previous estimates of 50-60% of anaemia attributable to iron deficiency are found.^89^ A Cochrane review of trials of antenatal iron-folic acid supplementation (a current WHO policy)^90^ found the risk reduction in anaemia in pregnancy to be 70%, suggesting that a high burden of iron deficiency, resulting from increased requirements of pregnancy and poor dietary availability, could be a major underlying cause of anaemia during pregnancy. Other causes include haemoglobinopathies, infections, and other nutritional deficiencies, such as that of vitamin A, folic acid (part of standard of care with iron), and vitamin B12. When examining wealth inequity in the prevalence of anaemia in pregnancy (appendix p 18), many high burden countries rank the highest, suggesting underlying socioeconomic, dietary, and health drivers, which could be overcome to address anaemia in women and reduce the equity gap.

### Maternal micronutrient deficiencies

Globally, there is a need to address inadequate quality of diets, especially among women of reproductive age, including pregnant and lactating women. Yet, the burden of vitamin and mineral deficiencies caused by inadequate diets, especially availability and intakes of animal food sources rich in bioavailable micronutrients, is difficult to estimate due to a scarcity of data. Micronutrient deficiencies have been mapped^91^ with the burden of anaemia, serum retinol, and stunting, and modelled estimates of global food supply and intakes, but measurement of biochemical indicators of micronutrient deficiencies in populations would be preferable.^92^ A review of data^93^ (appendix p 19) by use of biochemical indicators found clear evidence that women have several deficiencies of essential micronutrients during their reproductive lives. Biochemical analyses of nutrient indicators from two trials in Nepal and Bangladesh found that almost 80% of women had at least two micronutrient deficiencies at the outset of pregnancy.^94,95^ Although daily supplementation providing recommended dietary allowances of 15 nutrients, compared with standard supplementation with iron and folic acid, reduced the rate of adverse birth outcomes,^96,97^ restoration of micronutrient sufficiency was not fully met,^95,98^ largely due to the increased requirements in pregnancy and the large gap in nutrient needs that chronically inadequate diets fail to meet.

A review of national nutrition surveys in LMICs showed that women of reproductive age frequently had a high percentage of deficiency in vitamins B12 and D, iodine, and zinc.^93^ Insufficient folate intake in women was estimated to result in 260 100 neural tube defects, including 57 800 stillbirths and 117 900 deaths among livebirths in 2015; no time trend data are available.^99^

There is a dearth of data on maternal micronutrient deficiency from Africa, a region in which declining trends in micronutrient density in the food supply are reported.^92^ Globally, the supply and intake of micro-nutrients have increased over time due to growth in both overall food supply and micronutrient density. However, this growth is not directly linkable to intakes in women, who, in many LMICs, might have suboptimal diets due to intrahousehold food distribution inequities associated with gender bias, among other factors.

## Data needs

National and subnational governments, bilateral and global institutions, and civil society use data on nutrition indicators for purposes including advocacy, strategy development, monitoring, and evaluation. National household surveys constitute the main data source for nutrition indicators, and for analyses such as the ones discussed (appendix pp 1-5). Between 2000 and 2009, the number of such surveys was 136, which increased to 219 since 2010, but not all national surveys provide data on all outcomes. Also, survey results are not always available at levels of disaggregation that support local strategy or accountability, and tend to be focused on low-income countries, and, to a lesser extent, on lower-middle-income countries. Although providing data on anthropometry and intervention coverage for women and children, very few surveys collect biological samples. On the basis of the review reported in this Series paper, the main data gaps relate to biomarkers for micronutrient status, particularly for women, and to fetal growth, including birthweight and gestational age assessments. The sparsity of data prevents accurate mapping of the global burden of micronutrient deficiencies in women despite their substantial contribution to reproductive health and productivity; renewed efforts and funding are needed to fill this vast data gap.

In many countries, pregnant women and children attending health services undergo anthropometric examinations and answer questions on diet. Yet, coverage and quality of such data are often suboptimal. Investments in such nutrition data systems are essential to ensure that countries and subnational units have access to the data needed to monitor progress and plan strategic actions to address malnutrition.

## Conclusion

The prevalence of maternal and child undernutrition remains unacceptably high, particularly in low-income countries and among the poorest households in middle-income countries. Maternal and child undernutrition continues to represent an important component of the double burden of malnutrition, sharing causes and consequences with overweight and obesity among children and adults.

Obtaining more information on these conditions at both national and subnational levels is a priority because it could enable targeting of interventions, understanding programme outcomes, and enhance accountability. More forceful efforts to address the unfinished agenda of maternal and child undernutrition are needed for countries to reach the World Health Assembly nutrition targets and the ambitious Sustainable Development Goals.

Our Series paper shows that progress in reducing undernutrition in women and children has been variable. Progress has been made in terms of reducing stunting in children and underweight in women, and in tackling iodine deficiency. Wasting prevalence has been reduced in some countries, but not in others. Exclusive breast-feeding rates, but not partial breastfeeding after age 12 months, have improved slightly. Anaemia in children and women remains nearly unchanged, as does low birthweight and zinc deficiency. Even for indicators that show improvement at global scale, specific regions or countries have made little progress. Socioeconomic inequalities between and within countries remain largely unabated and continue to curtail the rate of progress at a global scale.

Despite huge data gaps, we make a case for improving women’s nutrition, both for women’s health and for optimal fetal growth, with consequences for improved growth in childhood and for the next generation. Programmatic action needs to take a life course approach. For child undernutrition, when known interventions are delivered prospectively across each life stage, crucially from conception to age 2 years and for older children, their cumulative benefit is most likely to yield the highest gains in nutritional status and reductions in wasting, stunting, and micronutrient deficiencies. When appropriate, screening and targeting the most in need and those most likely to benefit, or geographical targeting, can result in greater improvements in nutritional status and probably increase the cost-effectiveness of nutrition interventions. The analyses of inequalities call for targeted interventions to narrow the gap.

The modest gains in selected indicators of maternal and child undernutrition are under threat by the COVID-19 pandemic. Disruptions to the economy, employment, food systems, health and education services, and social protection schemes, among others, are predicted to increase the prevalence of wasting in children, underweight in women, low birthweight, and micronutrient deficiencies in the short run. The modest improvements reported here for some indicators of undernutrition might be rapidly eroded in the context of the pandemic, unless urgent actions are taken at global, national, and subnational levels.^100^

## Supplementary Material

Appendix

## Figures and Tables

**Table:** Prevalence of underweight, short stature, and anaemia among women aged 15-49 years in countries with data for 2000 and 2015

	Low-income countries		Middle-income countries		All LMICs	
	2000	2015	2000	2015	2000	2015
**Low BMI**						
Women aged 15−19 years*						
Mean	7.7%	7.2%	0.7%	0.7%	6.8%	6.5%
95% CI	6.5−8.9	5.9−8.5	0.2−1.2	0.2−1.2	5.6−8.0	5.2−7.7
Countries	30	30	10	10	40	40
Women aged 20−49 years†						
Mean	31.0%	16.0%	2.1%	1.7%	27.2%	14.2%
95% CI	26.7−35.4	14.1−17.8	0.9−3.2	0.6−2.8	22.5−31.8	12.1−16.3
Countries	30	30	10	10	40	40
**Low height**						
Women aged 15−19 years‡						
Mean	34.0%	25.6%	16.7%	8.4%	30.6%	22.7%
95% CI	28.9−39.1	21.1−30.2	8.1−25.4	2.2−14.6	25.8−35.4	18.5−26.9
Countries	30	30	11	11	41	41
Women aged 20−49 years§						
Mean	10.5%	8.1%	4.7%	2.4%	9.3%	7.0%
95% CI	8.7−12.4	6.4−9.7	1.1−8.3	0.0−5.3	7.5−11.0	5.4−8.5
Countries	30	30	11	11	41	41
**Anaemia**						
Women aged 15−49 years¶						
Mean	52.8%	49.5%	29.1%	25.8%	51.0%	47.6%
95% CI	49.1−56.4	45.3−53.7	21.7−36.4	16.4−35.1	47.0−55.1	43.1−52.2
Countries	19	19	5	5	24	24
**Anaemia during pregnancy**						
Pregnant women ‖						
Mean	57.0%	48.8%	29.3%	24.6%	55.0%	46.9%
95% CI	53.4−60.7	45.5−52.2	16.8−41.8	15.3−34.0	50.5−59.5	42.9−51.0
Countries	19	19	5	5	24	24

Data are from Demographic and Health Surveys (appendix pp 15-17). We refer to data collected from 1996 to 2005 as 2000 data, and data collected from 2010 to 2018 as 2015 data. BMI=body-mass index. LMICs=low-income and middle-income countries.

* Percentage of women below the median Z score of −2 for BMI for age (WHO standard).

† Percentage of women below 18·5 kg/m^2^.

‡ Percentage of women below the median Z score of −2 for height-for-age (WHO standard).

§ Percentage of women below 145 cm.

¶ Percentage of non-pregnant women whose haemoglobin count is less than 12·0 g/dL and pregnant women whose haemoglobin count is less than 11.0 g/dL.

‖ Percentage of pregnant women whose haemoglobin count is less than 11.0 g/dL.

## Key messages

- From 2000 to 2015, there were small improvements in linear growth for children younger than 5 years, but progress was slow for wasting and low birthweight
- The distributions of height-for-age and weight-for-height remain shifted towards undernutrition in low-income countries, where stunting and wasting persist as important public health problems
- New evidence shows that the incidence of stunting and wasting are highest during the first 6 months of life, reinforcing the need to focus on the first 1000 days from conception to age 2 years
- Socioeconomic inequalities persist as a major distal determinant of undernutrition in women and children, as shown by between-country and within-country analyses
- Regarding micronutrient deficiency in young children, vitamin A status has improved in east Asia, but persists at high rates in south Asia and in Africa. Zinc deficiency affects one in every two children in the few countries with information. Anaemia remains highly prevalent among women and young children
- Investments to reduce undernutrition in women are important not only for women’s own health but also for the health and nutrition of their children, as new evidence reinforces the link between nutrition of mothers and reproductive outcomes
- There have been positive yet small reductions in the prevalence of low body-mass index among women of reproductive age
- The growing availability of anthropometric data for women and young children has allowed progress to be monitored and interventions to become more focused, but the scarcity of information on micronutrient status needs to be addressed
