# Supplementary material for: Revisiting maternal and child undernutrition in low-income and middle-income countries: variable progress towards an unfinished agenda
Source: Lancet. Author manuscript; Available in PMC 2022 Jul 28. (PMC7613170; doi:10.1016/S0140-6736(21)00394-9)
Supplement: Appendix [file EMS151074-supplement-Appendix.pdf]

THE LANCET

Supplementary appendix

This appendix formed part of the original submission and has been peer reviewed. We post it as supplied by the authors.

Supplement to: Victora CS, Christian P, Vidaletti LP, Gatica-Domínguez G, Menon P, Black RE. Revisiting maternal and child undernutrition in low-income and middle-income countries: variable progress towards an unfinished agenda. *Lancet* 2021; published online March 7. http://dx.doi.org/10.1016/S0140-6736(21)00394-9.

Supplementary materials

Analyses of time trends in anthropometry of children under the age of five years

The national surveys database at the International Center for Equity in Health (Federal University of Pelotas, Brazil) includes over 400 nationally representative demographic, health and nutrition surveys from 100 countries, carried out from 1995 onwards (www.equidade.org). The database is maintained by the Equity Technical Working Group of the Countdown to 2030 Initiative, funded by the Bill and Melinda Gates Foundation and the Wellcome Trust.

For 50 countries, two surveys with anthropometric measurements on children under five years of age were available, one that had been carried out from 1996-2005 (referred to as "circa 2000" surveys), and another carried out on 2010 or later ("circa 2015" surveys). The full list of countries and surveys is available in the Table below. Thirty-one countries were classified as low income, and 19 as middle income according to their status in 2000 (https://datahelpdesk.worldbank.org/knowledgebase/articles/906519-world-bank-country-and-lending-groups).

Most surveys are either Demographic and Health Surveys (DHS) or Multiple Indicator Cluster Survey (MICS). Further information on the methodology employed by these surveys is available elsewhere: DHS (https://dhsprogram.com/what-we-do/survey-Types/dHs.cfm) and MICS (http://mics.unicef.org/). Both types of survey programs are highly comparable in terms of sampling and questionnaires.[1,2] In Peru, data for 2015 were obtained from the ENDES (https://proyectos.inei.gob.pe/endes/) and in Ecuador the 2015 ENSANUT (https://www.ecuadorencifras.gob.ec/encuesta-nacional-de-salud-salud-reproductiva-y-nutricion-ensanut-2012/) was analyzed.

All surveys employed standard methodology to measure children's weights, lengths (for children aged 0-23 months of age) or heights (for children aged 24-59 months). Z scores of height or length for age, and of weight for length or height were calculated using the World Health Organization Child Growth Standards (https://www.who.int/childgrowth/en/).

All surveys provided the information for calculating asset indices, obtained from information on household appliances, characteristics of the building materials, presence of electricity, water supply and sanitary facilities, among other variables[3,4]. Because relevant assets may vary in urban and rural households, separate principal component analyses are carried out in each area, which are later combined into a single score using a scaling procedure to allow comparability between urban and rural households. This score is then divided into quintiles.[5] Analyses by wealth quintile were pooled across low-income and middle-income countries, and are presented in Figure 4 in the body of the article.

Data analyses were carried out with Stata 15.0 (Statacorp, College Station, TX, USA). All analyses took into account the complex survey design. For all analyses showing results from multiple countries, national results were pooled using their estimated underfive population in years 2000 and 2015 as weights (https://www.who.int/nutgrowthdb/estimates2018/en/).

The curves in Figure 2 in the article, and similar bell-shaped curves in the Supplementary Materials, were smoothed using kernel density estimation or KDE.[6] For figures 3 and 4 in the article, and similar figures of anthropometry by age in months in the Supplementary Materials, the Stata lpolyci command was used for smoothing the curves by age and calculating 95% confidence limits.(https://www.stata.com/manuals13/rlpoly.pdf)

Table SM1. List of surveys included in the analyses, showing sample sizes, means and standard deviations for HAZ (height or length for age) and WHZ (weight for length or height).

Country	Survey	Year	HAZ			WHZ		
			Children	Mean	SD	Children	Mean	SD
[b] **Albania**	MICS	2000	1,082	-0.66	1.84	1,079	0.56	1.65
	DHS	2015	2,485	-0.39	1.55	2,455	0.81	1.32
[a] **Armenia**	DHS	2000	1,533	-0.82	1.39	1,523	0.80	1.28
	DHS	2015	1,581	-0.22	1.68	1,564	0.58	1.43
[a] **Bangladesh**	DHS	2000	4,706	-2.33	1.54	4,706	-0.97	1.33
	DHS	2015	7,235	-1.53	1.38	7,207	-0.89	1.16
[a] **Benin**	DHS	2000	3,866	-1.42	1.58	3,866	-0.30	1.30
	DHS	2015	12,013	-1.47	1.34	12,009	-0.28	1.08
[a] **Burkina Faso**	DHS	2000	3,686	-1.56	1.74	3,686	-0.68	1.33
	DHS	2015	6,948	-1.38	1.64	6,888	-0.64	1.42
[a] **Cambodia**	DHS	2000	3,517	-1.95	1.72	3,517	-0.79	1.41
	DHS	2015	4,906	-1.41	1.42	4,897	-0.63	1.21
[a] **Cameroon**	DHS	2000	3,183	-1.38	1.71	3,183	0.24	1.36
	MICS	2015	6,726	-1.19	1.64	6,615	0.22	1.27
[a] **Chad**	DHS	2000	5,505	-1.60	1.87	5,505	-0.69	1.39
	DHS	2015	11,036	-1.61	1.98	11,086	-0.63	1.37
[b] **Colombia**	DHS	2000	4,199	-1.01	1.17	4,199	0.40	1.01
	DHS	2015	17,824	-0.85	1.15	17,804	0.31	1.00
[b] **Congo, Republic**	DHS	2000	3,893	-1.05	1.82	3,893	0.04	1.43
	MICS	2015	8,729	-0.99	1.73	8,699	-0.21	1.26
[a] **Côte d'Ivoire**	DHS	2000	1,489	-1.19	1.54	1,489	-0.13	1.30
	MICS	2015	8,949	-1.09	1.43	8,958	-0.33	1.09
[b] **Dominican Republic**	DHS	2000	3,732	-0.79	1.34	3,732	0.24	1.12
	DHS	2015	3,691	-0.35	1.26	3,666	0.26	1.22
[b] **Ecuador**	RHS	2000	5,215	-1.19	1.30	5,187	0.10	1.03
	ENSANUT	2015	8,376	-1.19	1.35	8,267	0.39	1.16
[b] **Egypt**	DHS	2000	12,379	-1.02	1.81	12,379	0.50	1.48
	DHS	2015	14,678	-0.40	2.16	14,265	0.17	1.80
[b] **El Salvador**	RHS	2000	6,650	-1.24	1.19	6,575	-0.07	0.97
	MICS	2015	7,225	-0.82	1.13	7,194	0.25	1.15
[a] **Ethiopia**	DHS	2000	8,574	-2.07	1.69	8,574	-0.68	1.27
	DHS	2015	8,931	-1.37	1.75	8,925	-0.60	1.28
[b] **Gabon**	DHS	2000	3,443	-1.20	1.55	3,443	0.12	1.18
	DHS	2015	4,143	-1.02	1.59	4,115	0.17	1.28

Country	Survey	Year	HAZ			WHZ		
			Children	Mean	SD	Children	Mean	SD
[a] **Gambia**	MICS	2000	6,388	-1.03	1.47	6,382	-0.47	1.10
	DHS	2015	3,626	-1.07	1.65	3,562	-0.57	1.32
[a] **Ghana**	DHS	2000	2,632	-1.35	1.59	2,632	-0.50	1.24
	DHS	2015	3,053	-0.98	1.32	3,041	-0.26	1.09
[b] **Guatemala**	DHS	2000	3,897	-2.27	1.43	3,897	0.38	1.20
	DHS	2015	12,280	-1.88	1.20	12,268	0.38	1.00
[a] **Guinea**	DHS	2000	2,958	-1.15	1.74	2,958	-0.32	1.40
	MICS	2015	7,064	-1.29	1.73	7,038	-0.29	1.31
[a] **Haiti**	DHS	2000	5,534	-1.23	1.45	5,534	-0.11	1.19
	DHS	2015	5,614	-0.94	1.45	5,588	-0.01	1.13
[b] **Honduras**	RHS	2000	5,640	-1.38	1.25	5,628	-0.10	0.91
	DHS	2015	10,953	-1.22	1.24	10,937	0.29	1.04
[a] **India**	DHS	2000	41,306	-1.71	1.67	41,306	-0.91	1.33
	DHS	2015	240,098	-1.42	1.77	236,707	-0.94	1.43
[b] **Jordan**	DHS	2000	5,608	-0.71	1.22	5,608	0.17	1.07
	DHS	2015	6,410	-0.48	1.22	6,394	0.25	1.11
[b] **Kazakhstan**	DHS	2000	569	-0.73	1.39	569	0.26	1.16
	MICS	2015	5,263	0.00	1.59	5,189	0.44	1.28
[a] **Kenya**	DHS	2000	4,722	-1.34	1.62	4,722	-0.05	1.35
	DHS	2015	20,750	-1.17	1.47	20,707	-0.13	1.20
[a] **Kyrgyzstan**	MICS	2000	2,898	-0.79	1.43	2,904	0.31	1.18
	MICS	2015	3,436	-0.62	1.29	3,430	0.48	1.08
[a] **Lesotho**	DHS	2000	1,373	-1.76	1.57	1,373	0.19	1.36
	DHS	2015	1,909	-1.53	1.37	1,892	0.35	1.23
[b] **Macedonia**	MICS	2000	4,291	-0.27	1.64	4,261	0.57	1.36
	MICS	2015	1,317	0.03	1.39	1,297	0.58	1.29
[a] **Malawi**	DHS	2000	9,188	-1.98	1.71	9,188	0.21	1.40
	DHS	2015	5,144	-1.51	1.39	5,152	0.09	1.13
[a] **Mali**	DHS	2000	9,359	-1.62	1.83	9,359	-0.54	1.32
	MICS	2015	15,639	-1.27	1.67	15,697	-0.74	1.23
[b] **Moldova**	DHS	2000	1,300	-0.17	1.61	1,300	0.23	1.34
	MICS	2015	1,669	-0.17	1.23	1,660	0.25	1.08
[a] **Mongolia**	MICS	2000	3,351	-1.06	1.43	3,290	0.49	1.26
	MICS	2015	5,739	-0.61	1.23	5,729	0.81	1.02
[b] **Montenegro**	MICS	2000	864	0.52	1.64	850	0.62	1.42
	MICS	2015	1,366	0.33	1.71	1,322	0.91	1.50
[a] **Mozambique**	DHS	2000	8,059	-1.85	1.48	8,059	0.14	1.24
	DHS	2015	10,500	-1.60	1.65	10,501	0.24	1.36

Country	Survey	Year	HAZ			WHZ		
			Children	Mean	SD	Children	Mean	SD
[b] **Namibia**	DHS	2000	2,919	-1.13	1.50	2,919	-0.44	1.31
	DHS	2015	2,643	-0.98	1.53	2,608	-0.22	1.33
[a] **Nepal**	DHS	2000	6,194	-2.19	1.33	6,194	-0.73	1.07
	DHS	2015	2,362	-1.52	1.35	2,359	-0.64	1.11
[a] **Nigeria**	DHS	2000	4,282	-1.60	1.90	4,282	-0.28	1.46
	MICS	2015	27,625	-1.67	1.67	27,660	-0.53	1.20
[b] **Peru**	DHS	2000	14,877	-1.46	1.47	14,877	0.62	1.12
	ENDES	2015	23,313	-0.86	1.05	23,278	0.56	1.02
[a] **Rwanda**	DHS	2000	6,048	-1.72	1.74	6,048	0.01	1.43
	DHS	2015	3,816	-1.56	1.41	3,807	0.46	1.18
[a] **Senegal**	DHS	2000	2,847	-0.92	1.47	2,847	-0.47	1.19
	DHS	2015	10,746	-0.97	1.29	10,761	-0.65	1.07
[b] **Serbia**	MICS	2000	3,403	0.06	1.76	3,365	0.46	1.48
	MICS	2015	2,406	0.41	1.59	2,363	0.58	1.39
[a] **Sierra Leone**	MICS	2000	4,327	-1.51	1.89	4,315	-0.38	1.36
	MICS	2015	11,399	-1.35	1.59	11,435	-0.14	1.30
[a] **Tajikistan**	MICS	2000	4,152	-1.19	1.59	4,143	-0.27	1.32
	DHS	2015	5,868	-0.82	1.49	5,852	-0.08	1.27
[a] **Tanzania**	DHS	2000	5,212	-1.96	1.52	5,212	-0.19	1.34
	DHS	2015	8,974	-1.43	1.40	8,940	-0.08	1.18
[b] **Thailand**	MICS	2000	9,075	-0.47	1.35	8,981	-0.08	1.36
	MICS	2015	11,208	-0.63	1.34	11,138	0.01	1.42
[a] **Uganda**	DHS	2000	5,147	-1.76	1.50	5,147	0.10	1.23
	DHS	2015	4,414	-1.21	1.45	4,396	0.07	1.18
[a] **Zambia**	DHS	2000	5,517	-1.96	1.53	5,517	0.08	1.28
	DHS	2015	12,625	-1.55	1.68	12,573	0.01	1.36
[a] **Zimbabwe**	DHS	2000	2,647	-1.22	1.73	2,647	0.12	1.50
	DHS	2015	4,918	-1.20	1.39	4,880	0.21	1.23

Notes: (a) Low income countries as of the year of the first survey.
(b) Middle-income countries.

Figure SM1. Stunting and wasting prevalence around 2000 and 2015, within World Bank country income groups. Each dot is one country. Sources: Demographic and Health Surveys and Multiple Indicator Cluster Surveys.

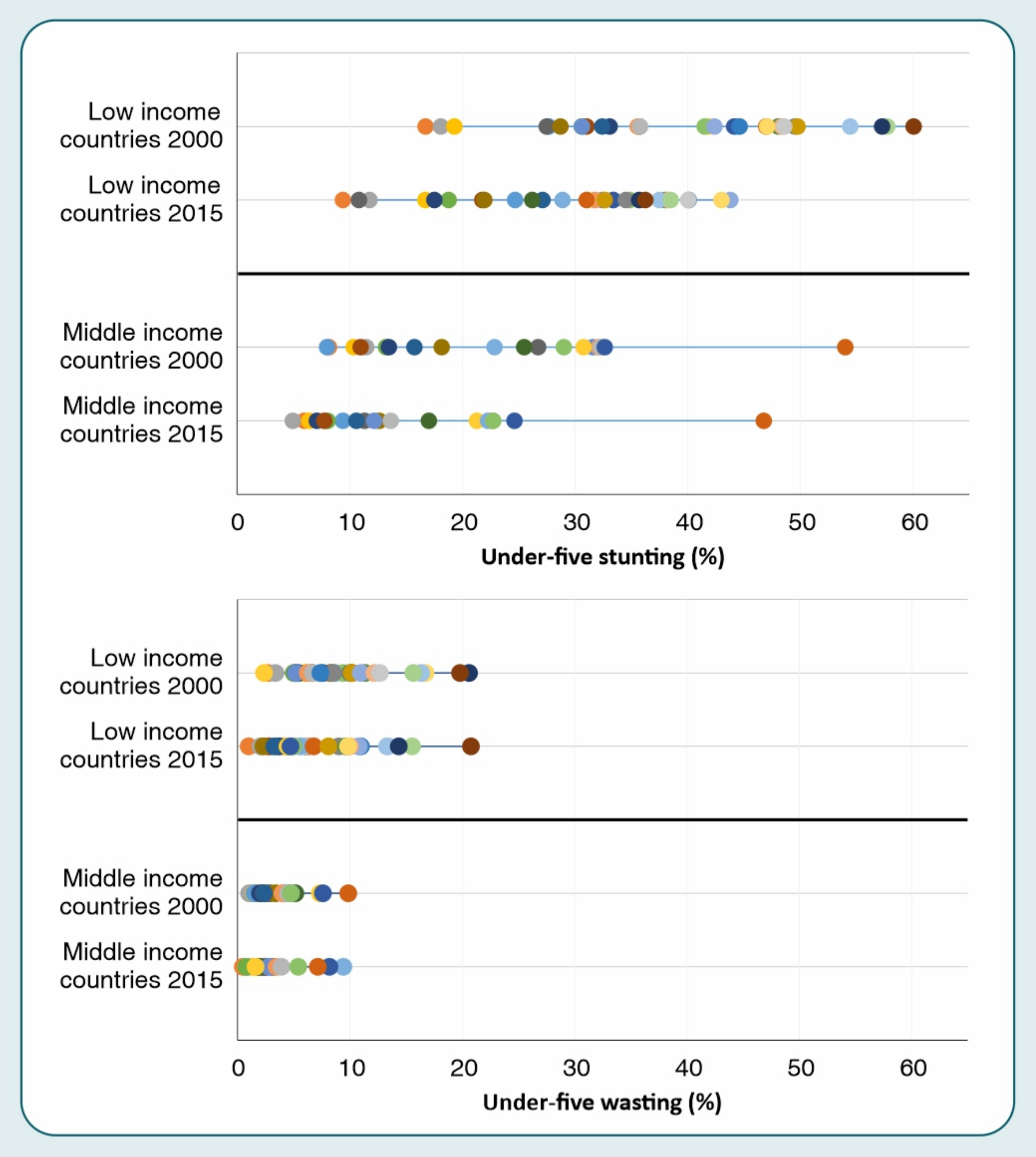

Within-country inequalities: persisting gaps between rich and poor children

Analyses of survey data by household wealth in LMICs, around 2015, allow the comparison of growth of children in the poorest and wealthiest quintiles, averaged across 31 low and in 19 middle-income countries.

The height for age distribution of children (Figure 1) in the wealthiest quintile in each country is closer to the WHO standard distribution than the distribution of children in the poorest quintiles, both in low-income (means -0.83 (SD 1.56) and -1.78 (SD 1.72), respectively) and in middle-income countries (means -0.30 (SD 1.80) and -1.07 (SD 1.60), respectively). In middle-income countries, children in the wealthiest quintile are already close to the WHO standard in terms of mean values. The SD values are well above 1.0, due to the combination of multiple populations from different countries.

For weight for height, data from low-income countries (Figure 3.1) show similar patterns to those observed for height for age, with children from the poorest quintile presenting lower weights for height than those in the richest quintile (means -0.82 (SD 1.38) and -0.43 (SD 1.38), respectively). The picture is rather different in middle-income countries as both extreme quintiles present average weights for height above the WHO median (means 0.20 (SD 1.29) and 0.31 (SD 1.58), respectively)., with an excess of children who are overweight, particularly those in the richest quintile.

Analyses of mean Z scores by age in months provide additional insights into the timing of growth faltering. These analyses are presented in Figure 3 in the body of the article. In low income countries, there are important differences in birth length between rich and poor children. Mean values of height for age suggest that the latter also present more intense and longer period of linear growth faltering. The differences in middle-income countries are in the same direction as in low-income countries, but less marked.

Weight for length at birth was similar for both rich and poor children in low income countries, but whereas mean values for the latter continue to fall during the first year of life, the average values for the wealthiest quintile increase from birth onwards. In middle-income countries both groups of children are born slightly above the WHO median, and mean values for poor children remain parallel to the standard, whereas the weights for height in the wealthiest quintile continues to increase during the first two years.

These analyses confirm the importance of social determinants of undernutrition and reveal major gaps between children from poor and wealthy families in terms of linear growth. For weight for height, differences were less marked but still present in low-income countries. Overweight, rather than wasting, has become the leading problem in middle-income countries.

Figure SM2. Height-for-age (top) and weight-for-height (bottom) distributions of children under five years of age in the poorest and wealthiest quintiles in 2015, compared to the WHO standard (light blue curves). The black vertical line corresponds to -2 Z-scores line (black lines). Analyses of 50 countries with two surveys.

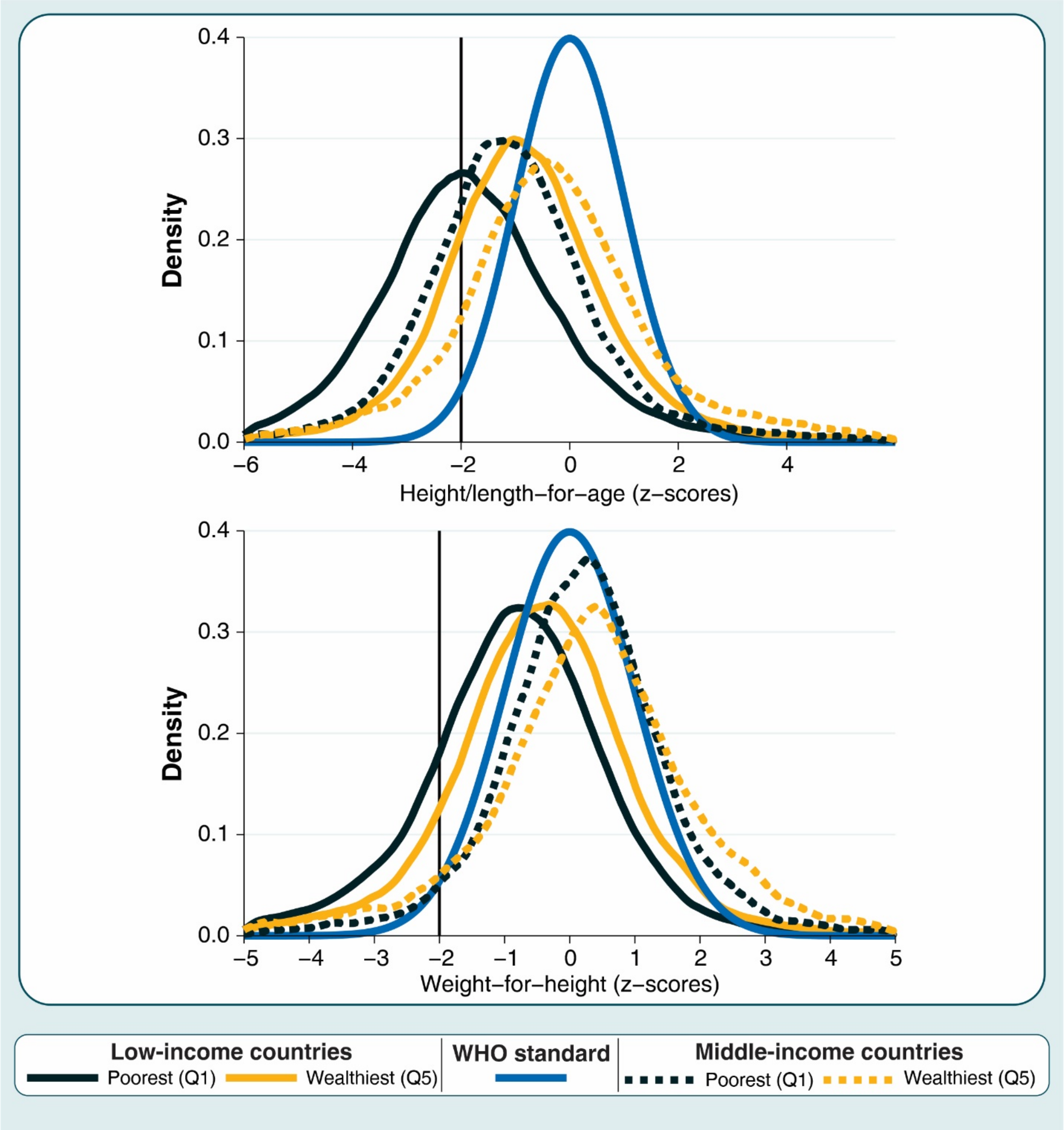

Child growth in India: A picture of transitions from 1998 to 2015

India has the world's largest burden of child undernutrition with about a third of the global burden of stunting and close to half the global burden of wasting. National surveys in India have used varying age groups in different surveys, making it difficult to assess long term trends. In particular, the 1998 survey was restricted to children under three years, whereas more recent surveys included all underfives. We thus analyzed data for children under the age of three years to examine trends in prevalence, shifts in distributions and age-specific patterns of height-for-age z-scores and weight for height z-scores over time (Figures SM3 and SM4).

The burden of stunting among children under-three declined from 50% in 1998 to 36% in 2015 with greater reductions in both stunting and severe stunting between 2005 and 2015 than in the period preceding that. Wasting, however, has barely changed over the same time, and severe wasting appears to have gone up in 2015. At the same time, the shifts in the overall distribution of child HAZ and WHZ are small, suggesting a large population-level prevalence of poor child growth. India has a long way to go to move the child growth distribution towards the WHO standard population distribution. The HAZ-by-age curves suggest improvements occurred mainly in the first few months of life between 1998 and 2005, following which the curves remain parallel. In 2015, however, there are differences both in early life *and* a divergence of the curve at older ages, suggesting improvements across the age spectrum among children surveyed in 2015-16.

Wasting shows the least change over time, unfortunately, and WHZ appears to be stable across age groups both in 1998 and 2005. However, in 2015, WHZ is higher in younger age groups and in older age groups, but lower during the period of complementary feeding (9-15 months), suggesting greater vulnerability.

India was classified by the World Bank as a low-income country (LIC) in 2000. Figures SM5-8, corresponding to similar figures in the body of the paper, show how India compares with other countries in this group. The distribution of height-for-ages and prevalence of stunting in Indian children are similar to those in other LICs, but there are marked differences in the distribution of weight-for-heights and wasting prevalence, with Indian children showing worse status than children from other LICs.

Lastly, Figure SM9 show that there is wide variability in the prevalence of stunting and wasting among Indian states, particularly in terms of wasting

Efforts to tackle stunting and wasting in India will need sustained effort for many years to come. The determinants of childhood growth in India are multi-faceted - poor maternal nutrition, poor complementary feeding practices, sanitation, access to services and poverty.[7,8] However, new case studies of subnational efforts that triggered greater reductions in stunting in some states compared to the national trends offer a range of lessons on what might work to accelerate improvements across India.[9]

India's political and administrative leadership has now embraced the challenge of malnutrition as one to tackle head on. An ambitious national nutrition "mission" launched by the Prime Minister of India in 2018 and built on a multisectoral strategy aims to accelerate actions using existing

large-scale program platforms, a national behavior change campaign, and efforts to strengthen multisectoral convergence.[10]

Table SM3. Height-for-age (top) and weight-for-height (bottom) distributions of Indian children under three years of age in 1998, 2005 and 2015, compared to the WHO standard (green curves). The black vertical lines correspond to the traditional cut off of -2 Z scores.

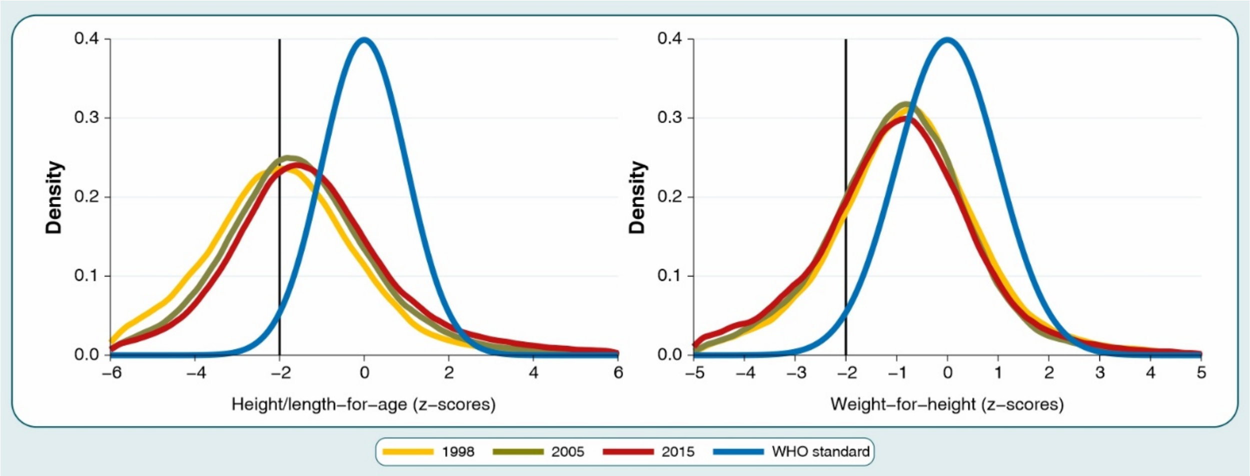

Figure SM4. Mean height-for-age (top) and weight-for-height (bottom) Z scores by age in months of Indian children under three years of age in 1998, 2000 and 2015, compared to the WHO standard median and -2 Z scores (black lines).

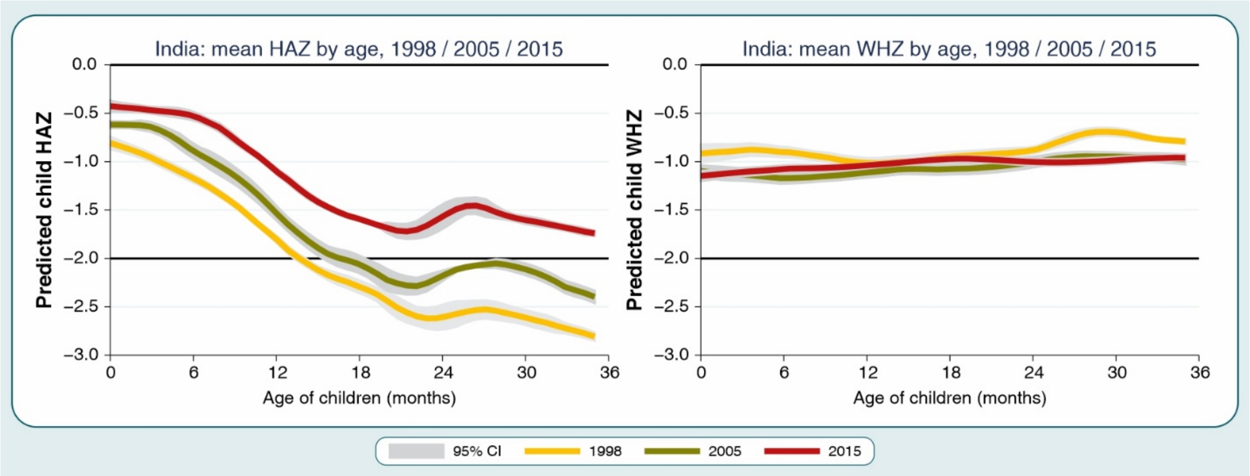

Figure SM5. Prevalence of stunting, wasting and co-occurrence of stunting and wasting. Comparison of India with other low-income countries in 2000 and 2015. Sources: Demographic and Health Surveys and Multiple Indicator Cluster Surveys.

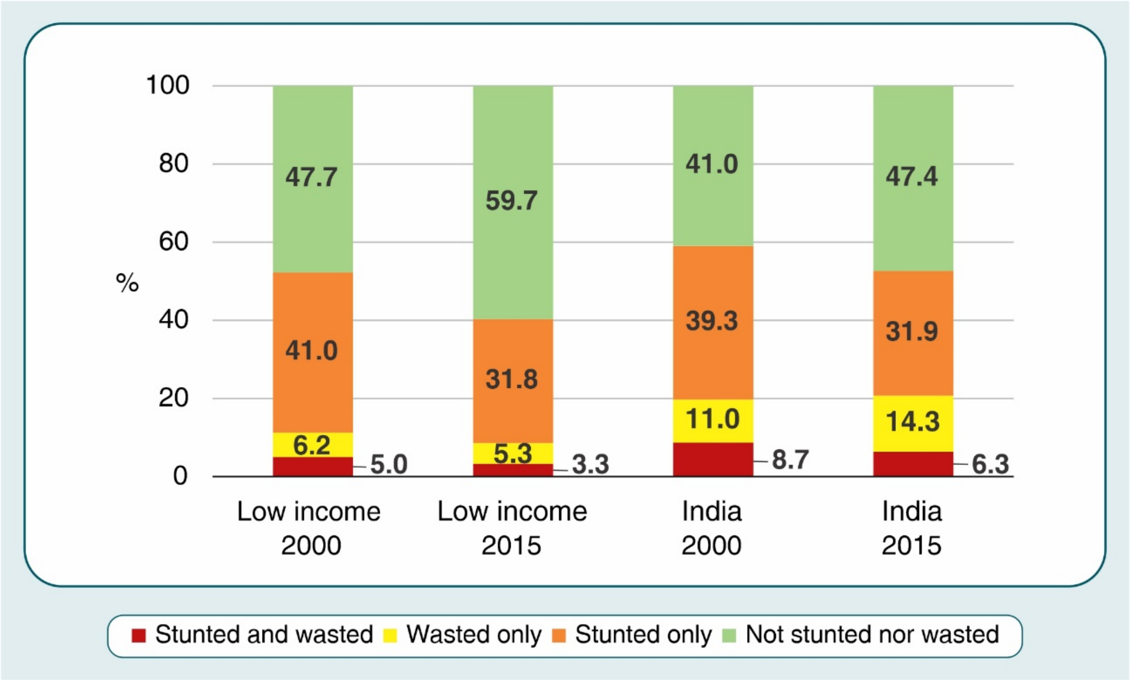

Figure SM6. Distribution of height-for-age and weight-for-height Z scores. Comparison of India with other low-income countries in 2000 and 2015. Sources: Demographic and Health Surveys and Multiple Indicator Cluster Surveys.

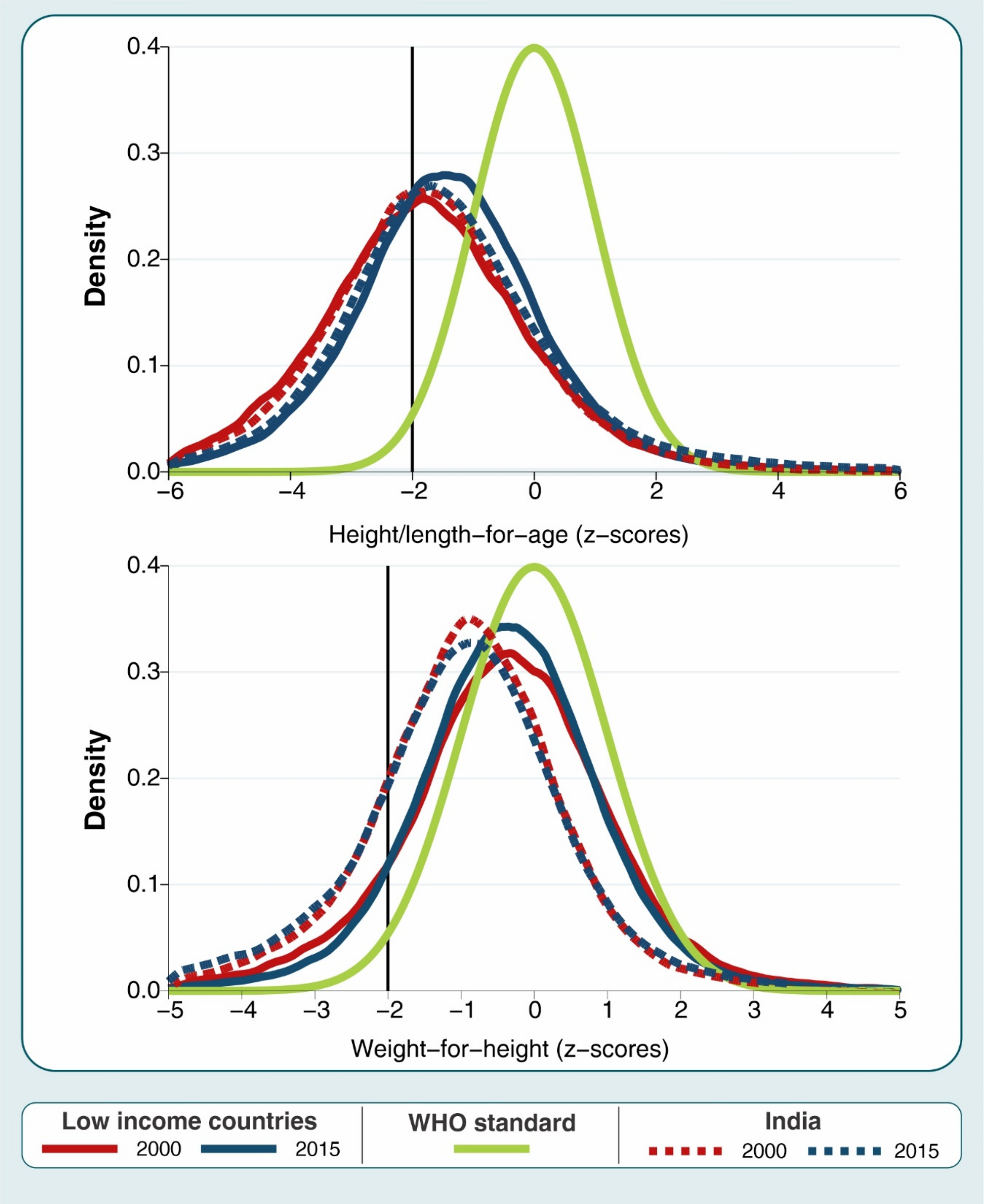

Figure SM7. Mean height-for-age and weight-for-height Z scores by age in months. Comparison of India with other low-income countries in 2000 and 2015. Sources: Demographic and Health Surveys and Multiple Indicator Cluster Surveys.

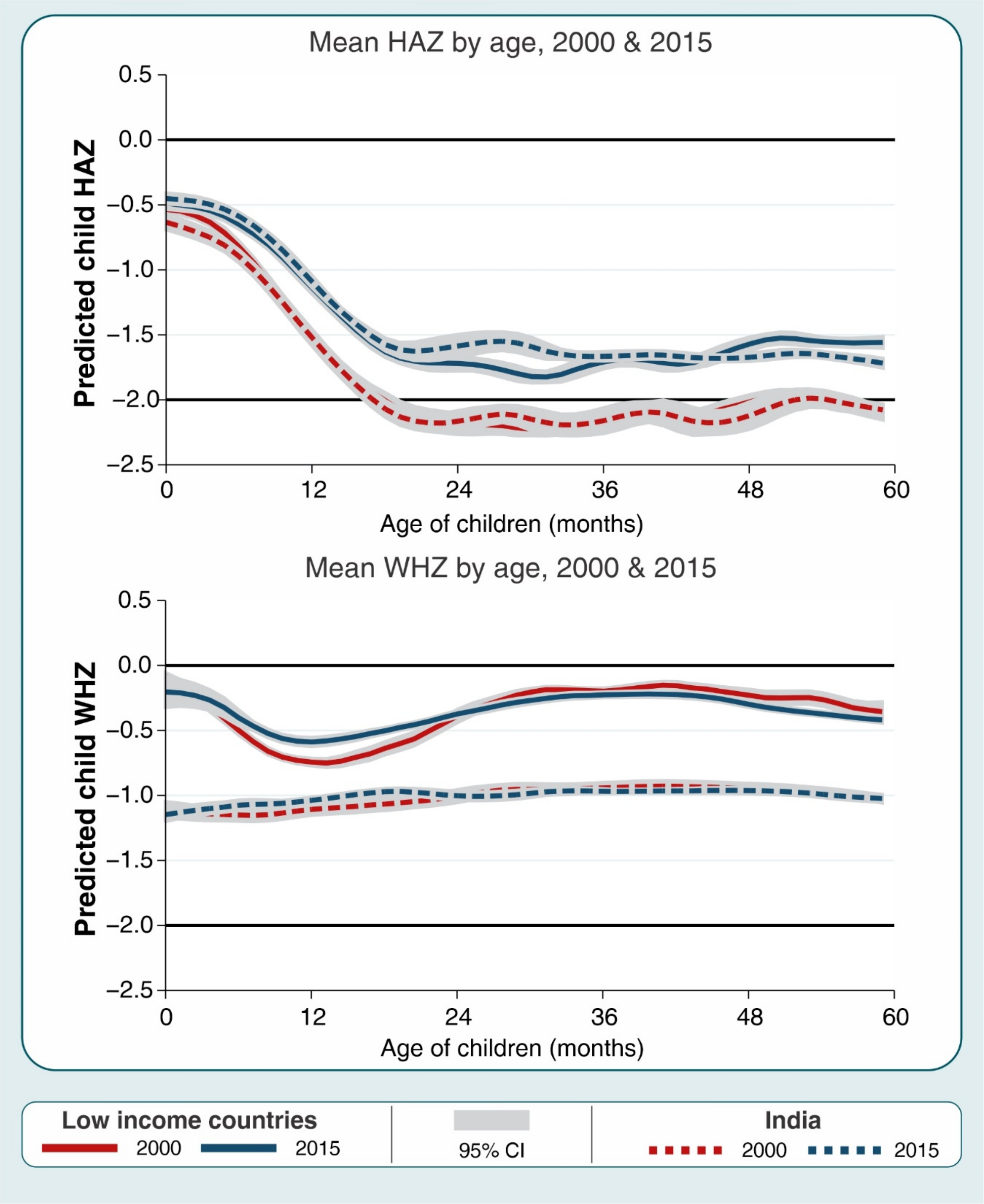

Figure SM8. Stunting (top) and wasting (bottom) prevalence around 2000 and 2015, within World Bank country income groups and Indian states. Each dot is one country or state. Sources: Demographic and Health Surveys and Multiple Indicator Cluster Surveys.

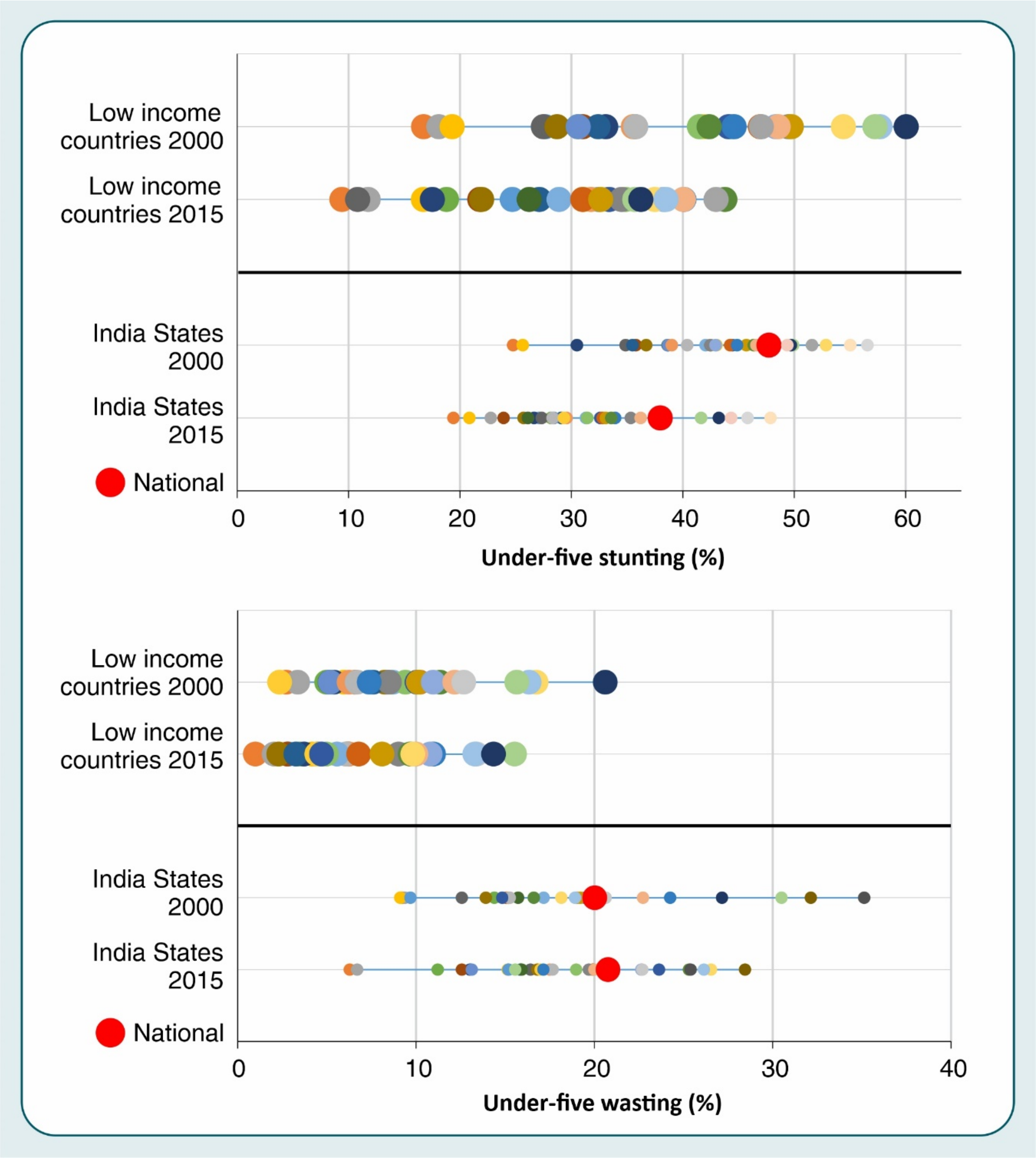

Anthropometric and anemia indicators for women, by country

Table SM2. List of surveys included in the analyses of women's undernutrition, showing low height, low BMI and anemia around 2000 and 2015. Source: Demographic and Health Surveys.

Country	Low height				Low BMI				Anemia[a]		Anemia in pregnancy[b]	
	Ages 15-19 y[c]		Ages 20-49 y		Ages 15-19 y[c]		Ages 20-49 y		Ages 15-49 y		Ages 15-49 y	
	2000	2015	2000	2015	2000	2015	2000	2015	2000	2015	2000	2015
**Armenia**	5.9	10.1	1.1	0.5	0.5	0.6	2.9	3.0	12.4	13.4	12.0	11.2
**Benin**	13.0	9.9	1.1	1.1	1.6	1.3	14.9	8.4	64.3	57.7	72.7	68.4
**Burkina Faso**	2.4	4.4	0.3	0.4	0.5	3.3	13.4	13.7	53.7	48.8	68.3	58.2
**Bangladesh**	46.1	38.3	17.0	12.6	12.5	5.5	52.4	17.2	-	-	-	-
**Brazil**	13.4	6.3	3.7	0.8	-	-	-	-	-	-	-	-
**Cote d'Ivoire**	5.2	8.6	0.8	0.9	1.5	1.6	7.7	5.4	-	-	-	-
**Cameroon**	5.5	5.8	1.2	0.5	2.4	0.9	7.2	4.8	44.9	39.7	50.9	39.4
**Congo Brazzaville**	18.3	6.9	4.2	1.4	2.4	3.0	10.8	11.4	57.0	54.2	69.8	58.4
**Colombia**	20.9	9.2	5.5	3.8	0.9	1.2	2.9	2.8	-	-	-	-
**Comoros**	29.1	9.9	3.7	2.4	2.9	1.5	10.1	5.0	-	-	-	-
**Dominican Republic**	8.8	3.3	2.9	1.6	1.2	3.3	5.6	4.4	-	-	-	-
**Egypt**	9.2	4.4	1.2	0.5	0.0	0.1	0.6	0.2	27.7	25.2	23.6	20.6

**Ethiopia**	18.7	11.6	2.5	2.2	10.6	5.1	27.3	20.5	26.6	23.6	30.6	29.1
**Gabon**	8.7	5.7	1.2	0.8	1.3	1.3	6.1	4.5	-	-	-	-
**Ghana**	9.6	6.2	0.6	0.8	1.2	1.6	11.0	4.3	44.6	42.4	61.1	44.6
**Guinea**	6.5	11.8	1.3	0.9	0.6	3.0	11.9	7.5	53.3	45.8	69.4	47.3
**Guatemala**	63.8	49.6	32.0	25.6	0.3	0.5	1.9	1.7	-	-	-	-
**Honduras**	24.7	21.8	10.1	8.3	0.9	1.1	2.7	2.8	18.7	15.1	21.4	18.8
**Haiti**	7.2	5.8	2.1	0.7	2.6	1.9	8.8	8.2	55.5	49.0	64.2	51.9
**India**	42.1	34.4	12.9	10.7	9.0	10.2	35.8	18.8	55.3	53.1	58.7	50.4
**Jordan**	5.3	2.0	1.2	0.5	0.0	0.0	2.2	1.2	29.1	43.5	37.0	32.2
**Kenya**	6.9	6.5	1.0	0.6	1.7	2.5	11.4	7.1	-	-	-	-
**Kyrgyzstan**	5.7	2.9	0.8	0.4	1.1	2.4	5.0	4.5	38.1	35.2	38.3	37.8
**Cambodia**	27.0	22.7	4.7	5.3	3.7	3.9	19.1	11.4	58.8	45.4	65.1	53.2
**Lesotho**	12.3	12.0	1.8	1.1	0.7	1.0	4.2	3.0	32.9	27.3	36.0	35.5
**Mali**	5.2	3.0	0.4	0.2	2.3	2.3	10.2	8.4	62.9	63.4	73.4	69.3
**Mozambique**	20.6	21.5	4.3	3.4	0.6	1.6	10.5	6.8	-	-	-	-
**Malawi**	18.0	15.0	2.4	2.3	1.6	1.1	6.6	5.6	44.3	32.7	47.3	45.1
**Niger**	5.5	5.9	0.4	0.6	2.6	6.3	20.1	12.4	-	-	-	-
**Nigeria**	24.5	10.5	6.2	1.0	5.5	4.5	15.8	9.0	-	-	-	-
**Nepal**	48.6	30.7	14.9	10.7	5.3	4.0	28.1	14.0	-	-	-	-

**Peru**	49.8	17.9	15.3	7.2	0.2	0.6	1.1	0.9	31.6	21.1	38.7	30.5
**Rwanda**	17.6	15.0	1.5	2.4	2.4	1.2	6.7	5.3	25.6	19.2	28.8	23.4
**Senegal**	2.0	2.1	0.3	0.2	7.0	8.2	12.8	18.1	59.1	54.1	70.6	62.7
**Chad**	3.3	3.9	0.2	0.3	5.2	4.9	20.8	17.2	-	-	-	-
**Togo**	10.1	8.0	0.9	0.6	2.3	0.9	10.7	5.8	-	-	-	-
**Turkey**	10.1	3.4	2.2	1.3	1.6	0.7	2.3	2.0	-	-	-	-
**Tanzania**	13.3	14.4	2.7	2.2	1.9	2.4	9.2	7.0	48.4	44.8	58.2	57.1
**Uganda**	12.7	8.8	1.8	1.1	1.6	0.9	9.6	7.4	36.7	31.7	47.1	38.2
**Zambia**	9.0	12.4	1.2	1.6	0.2	1.7	8.9	8.5	-	-	-	-
**Zimbabwe**	8.2	5.5	1.4	0.5	0.8	0.6	4.5	4.2	37.7	26.8	47.3	33.1

(a) % below the median -2 Z scores for BMI/age (WHO reference)

(b) % below 18.5 kg/m2

(c) % below the median -2 Z scores for height/age (WHO reference)

(d) % below 145 cm

(e) % non-pregnant women whose hemoglobin count is less than 12.0 g/dl plus number of pregnant women whose count is less than 11.0 g/dl

(f) % pregnant women whose count is less than 11.0 g/dl

Anemia prevalence by wealth quintile

For countries with a DHS since 2010, we estimated the prevalence of anemia among women of reproductive age. Figure 7 shows that prevalence is highest in the poorest wealth quintile, and lowest in the richest quintile, in about half of all countries with information. High-burden countries rank the highest in terms of inequality, suggesting underlying socioeconomic, dietary and health drivers that could be overcome to address anemia in women and reduce the inequity gap.

Figure SM9. Anemia prevalence among women aged 15-49 years by wealth quintile in the most recent Demographic and Health Survey (2010 or later). Countries are ordered according to the magnitude of inequality, according to the slope index of inequality.[11]

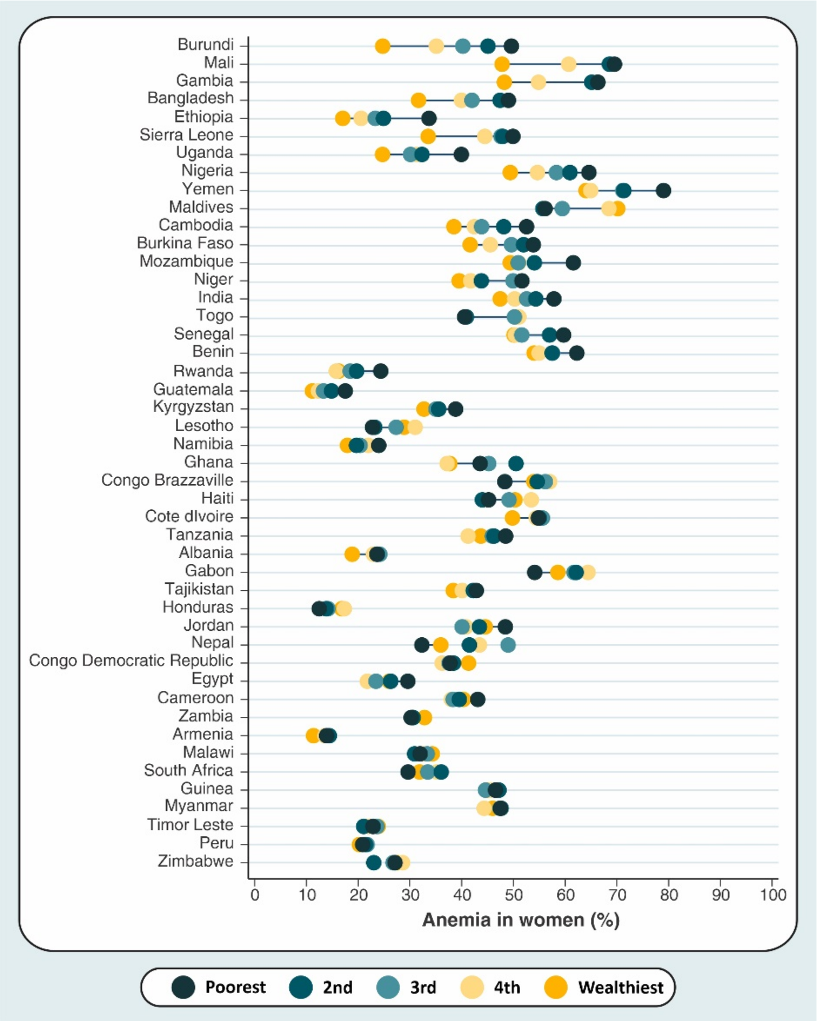

Evidence of micronutrient deficiencies in women of reproductive age

Data on micronutrient deficiencies in women from LMICs are scarce. A broad review published in 2019 presented the conclusions of a task force that assessed the prevalence of inadequate micronutrient intakes and adverse birth outcomes in these countries. [12] Figure 5 shows data on biochemical indicators of micronutrient deficiency, calculated from 52 national and regional surveys published between 2013 and mid-2017. Black dots indicate fewer than three countries in the region had data, and missing bars means no data were found for that micronutrient in the specific region.

This exercised confirmed the limited availability of data on this topic, but also provided clear evidence that women have a number of deficiencies of essential micronutrients during their reproductive lives.

Figure SM10. Regional estimates of micronutrient deficiencies and anemia as reported in women of reproductive age.

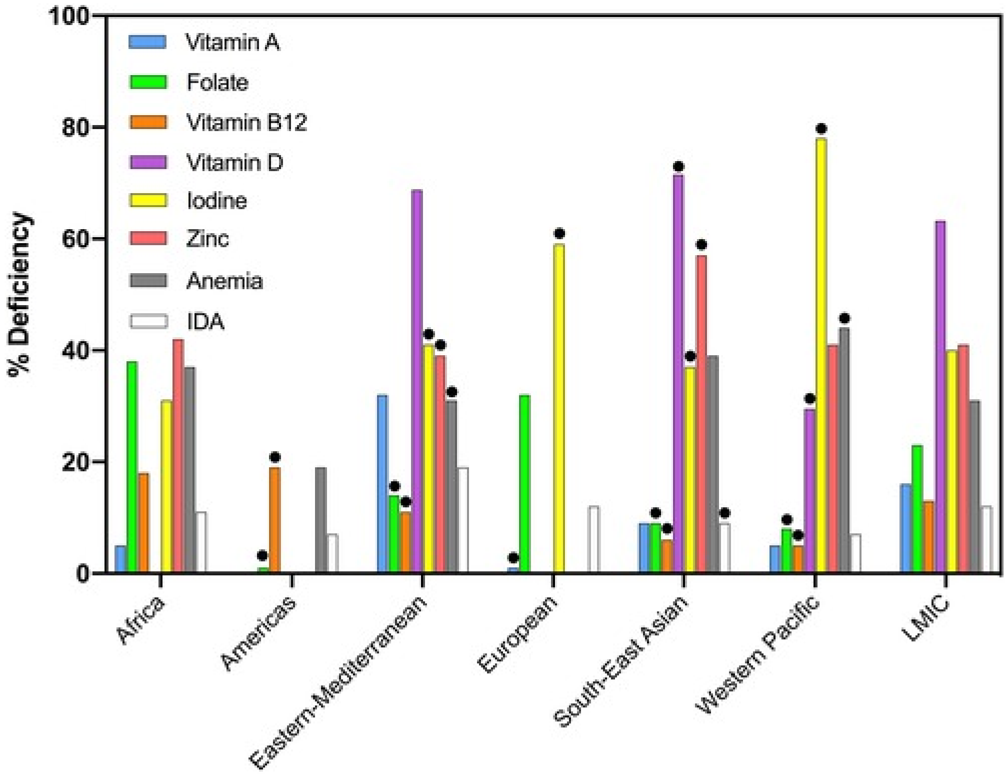

References

1. Corsi DJ, Neuman M, Finlay JE, Subramanian SV. Demographic and health surveys: a profile. *International journal of epidemiology* 2012; **41**(6): 1602-13.
2. Hancioglu A, Arnold F. Measuring coverage in MNCH: tracking progress in health for women and children using DHS and MICS household surveys. *PLoS medicine* 2013; **10**(5): e1001391-e.
3. Filmer D, Pritchett LH. Estimating wealth effects without expenditure data—or tears: an application to educational enrollments in states of India. *Demography* 2001; **38**(1): 115-32.
4. Rutstein SO, Johnson K, MEASURE OM. The DHS wealth index: ORC Macro, MEASURE DHS; 2004.
5. Rutstein SO. The DHS Wealth Index: Approaches for rural and urban areas. 2008.
6. Silverman B. Density Estimation for Statistics and Data Analysis. . London: Chapman & Hall; 1986.
7. Kim R, Rajpal S, Joe W, et al. Assessing associational strength of 23 correlates of child anthropometric failure: An econometric analysis of the 2015-2016 National Family Health Survey, India. *Soc Sci Med* 2019; **238**: 112374.
8. Menon P, Headey D, Avula R, Nguyen PH. Understanding the geographical burden of stunting in India: A regression-decomposition analysis of district-level data from 2015-16. *Maternal & child nutrition* 2018; **14**(4): e12620.
9. Kohli N, Avula R, van den Bold M, et al. What will it take to accelerate improvements in nutrition outcomes in Odisha? Learning from the past. *Global Food Security* 2017; **13**: 38-48.
10. Government of India. NITI Aayog 2018. Nourisig India: National Nutrition Strategy. New Delhi, 2017.
11. Harper S, Lynch J. Methods for Measuring Cancer Disparities: Using Data Relevant to Healthy People 2010 Cancer-Related Objectives. . Bethesda, MD: Division of Cancer Control and Population Sciences of the National Cancer Institute, 2005.
12. Bourassa MW, Osendarp SJM, Adu-Afarwuah S, et al. Review of the evidence regarding the use of antenatal multiple micronutrient supplementation in low- and middle-income countries. *Ann N Y Acad Sci* 2019; **1444**(1): 6-21.
